# Supplementary material for: Sleep waves in a large‐scale corticothalamic model constrained by activities intrinsic to neocortical networks and single thalamic neurons
Source: CNS Neurosci Ther. 2023 Apr 18;30(3):e14206. doi: 10.1111/cns.14206 (PMC10915987; doi:10.1111/cns.14206)
Supplement: Supplementary file 1 — Data S1: [file CNS-30-e14206-s001.pdf]

Sleep waves in a large-scale corticothalamic model constrained by activities intrinsic to neocortical networks and single thalamic neurons

Martynas Dervinis and Vincenzo Crunelli

Neuroscience Division, School of Bioscience, Cardiff University, Cardiff CF10 3AX, UK

SUPPLEMENTARY INFORMATION

Supplementary Figures	page 2
Supplementary Tables	page 22
Supplementary Methods	page 29
Supplementary Appendices	page 33
Supplementary References	page 47

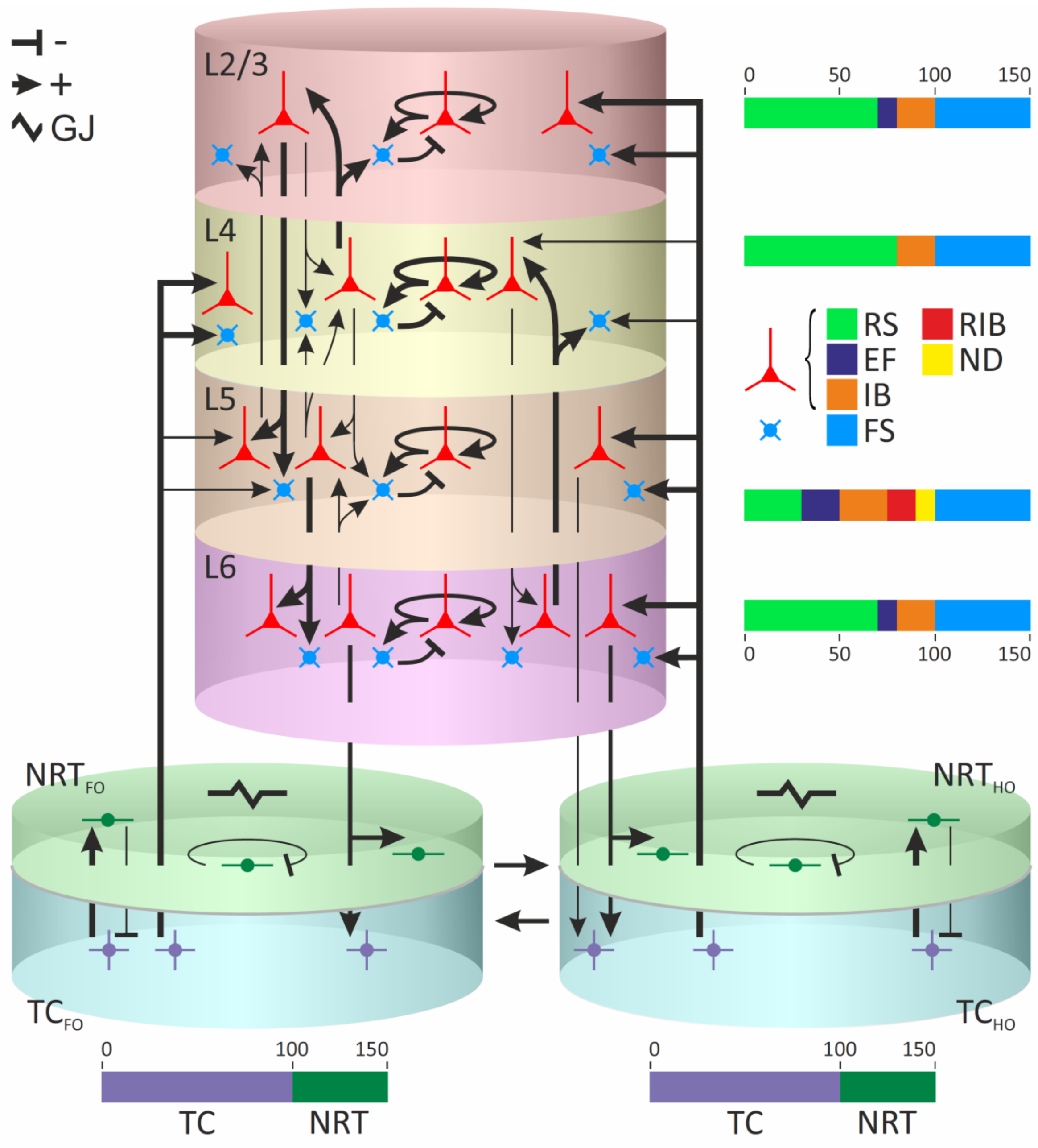

Figure S1. Corticothalamic model architecture.

The corticothalamic model consists of 900 neurons distributed in distinctly coloured layers of a single cortical column and two coloured-coded thalamic sectors, a first- and a higher-order sector. Each cortical layer contains 100 excitatory neurons (detailed neuronal populations are in the colour-coded bar on the right) and 50 fast-spiking (FS) inhibitory neurons. Thalamic cylinders (of both first-order and higher-order sector) contain 100 thalamocortical (TC) neurons and 50 nucleus reticularis thalami (NRT) neurons. Sharp and blunt arrows represent excitatory and inhibitory synaptic connections, respectively, with the line thickness indicating the synaptic connection strength (see actual values in Table S1). The lightning symbol indicates

gap junctions (GJ) that are present only between NRT neurons.

L2/3: cortical layer 2/3

L4: cortical layer 4

L5: cortical layer 5

L6: cortical layer 6

RS: regular spiking neuron

EF: early firing neuron

IB: intrinsically bursting neuron

RIB: repetitive intrinsically bursting neuron

ND: network driver neuron

FS: fast spiking neuron

TC_{FO}: thalamocortical neurons of first-order thalamic nucleus

TC_{HO}: thalamocortical neurons of higher-order thalamic nucleus

NRT_{FO}: nucleus reticularis thalami neurons connected to first-order nucleus,

NRT_{HO}: nucleus reticularis thalami neurons connected to higher-order nucleus.

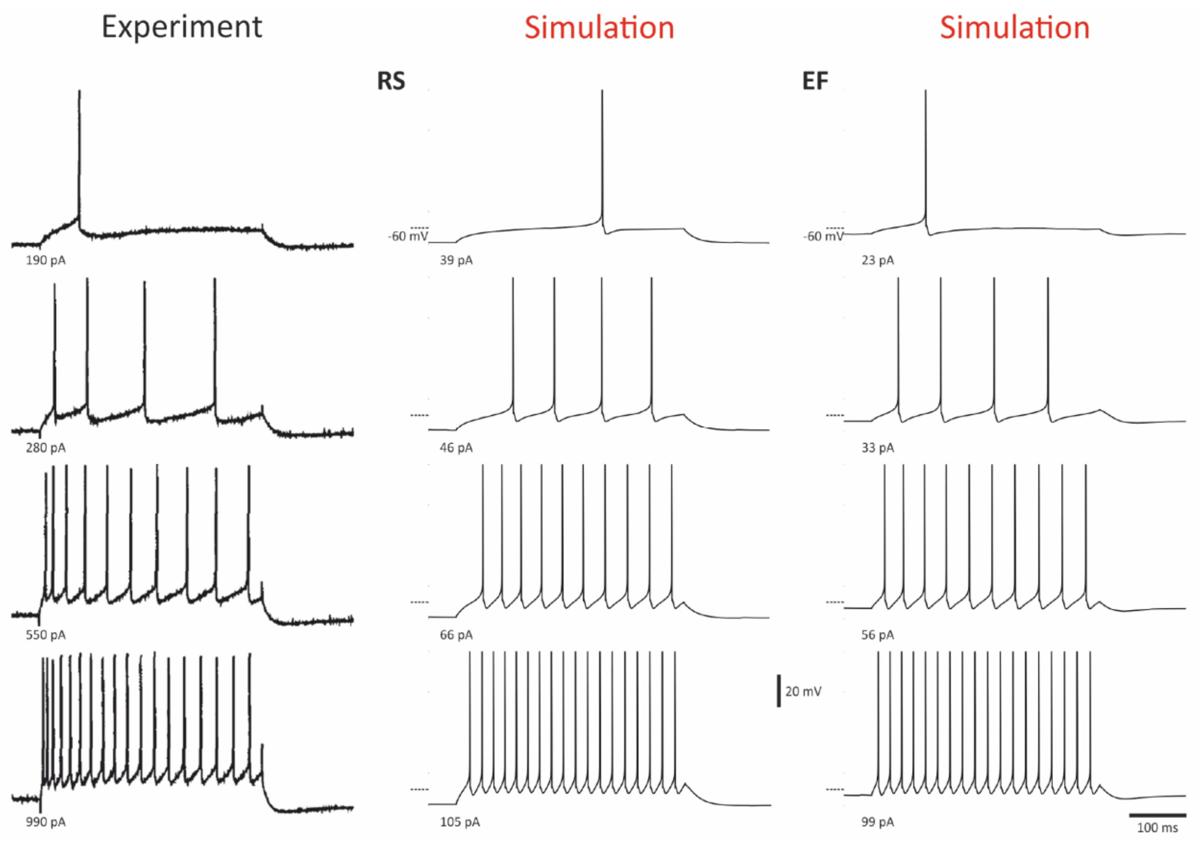

Figure S2. Experimental and simulated firing patterns of RS and EF neurons.

Intracellularly recorded firing patterns of a cat motor cortex RS neuron (Experiment, left column), simulated activity of the RS neuron model (Simulation, middle column) and simulated firing of an EF neuron (right column). Value of injected current is indicated below each trace. Note the more depolarized membrane potential of the EF neuron model compared to the RS neuron model, that was achieved by reducing g_{KL} in normal RS model neurons. Dashed line on the left of the traces indicates -60 mV. Experimental data are reproduced with permission from Chen, Zhang, Hu and Wu (1996)[1].

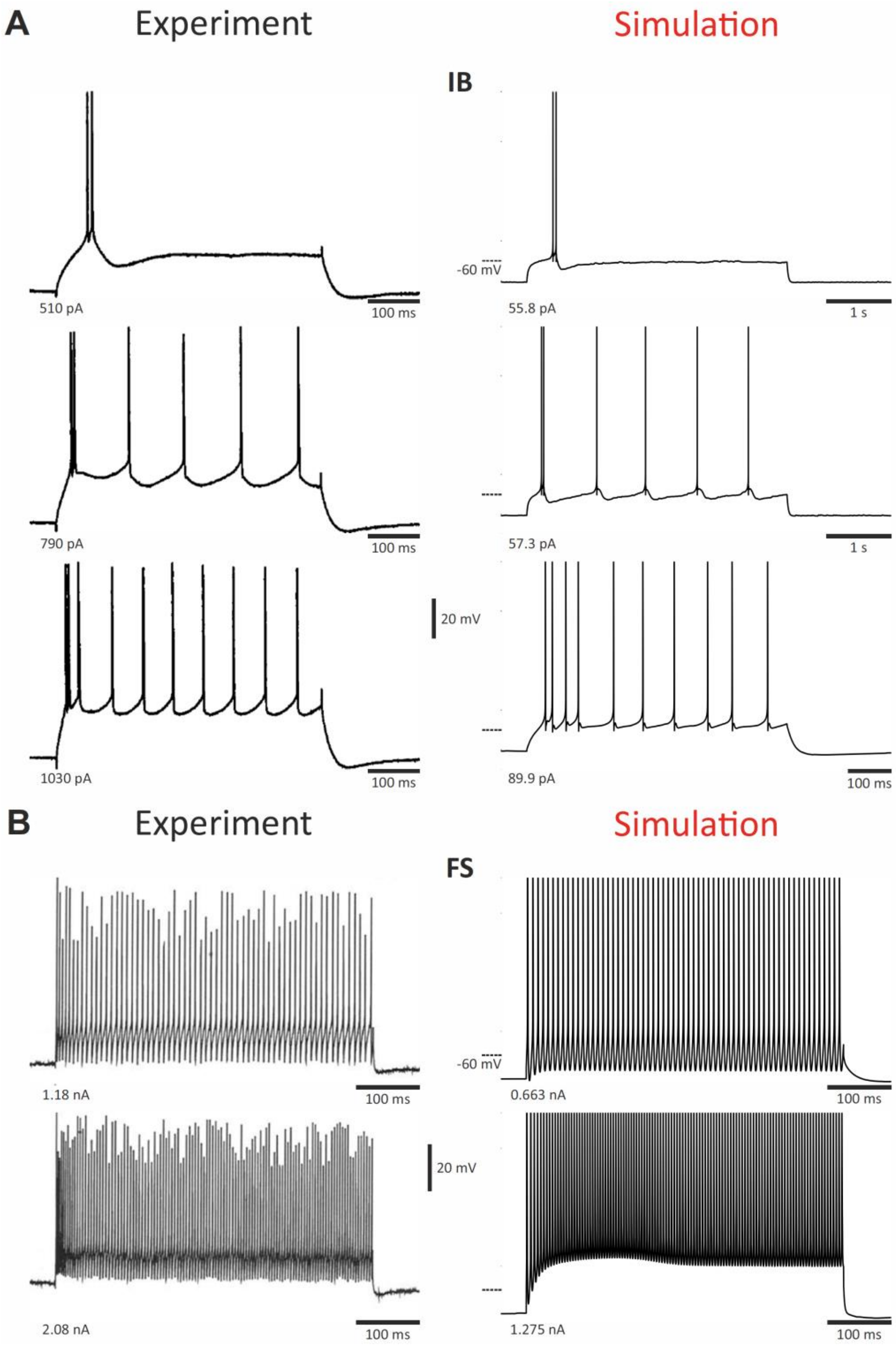

Figure S3. Experimental and simulated firing patterns of IB and FS neurons.

A, Intracellularly recorded firing patterns of a cortical RS neuron (Experiment) and simulated activity of the RS neuron model (Simulation). B, Intracellularly recorded firing patterns of a rat cortical FS neuron (Experiment) and simulated activity of the FS neuron model (Simulation). Value of injected current is indicated below each trace. Dashed line on the left of the traces indicates -60 mV. Experimental data are reproduced with permission from Chen, Zhang, Hu and Wu (1996)[1].

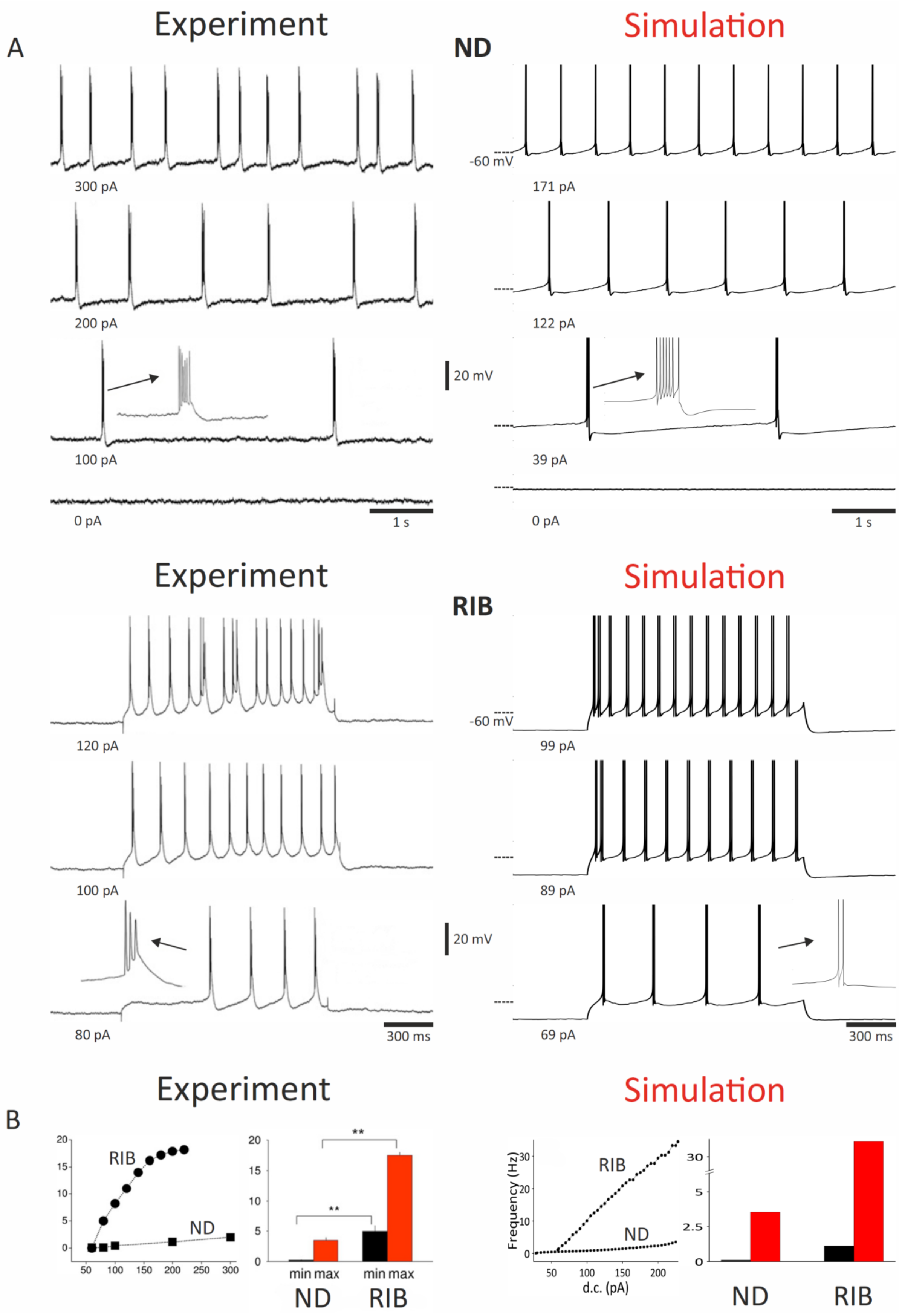

A
Experiment
Simulation
ND
-60 mV
300 pA
171 pA
200 pA
122 pA
20 mV
100 pA
39 pA
0 pA
1 s
Experiment
Simulation
RIB
-60 mV
120 pA
99 pA
100 pA
89 pA
20 mV
80 pA
69 pA
300 ms
B
Experiment
Simulation
RIB
ND
min max
Frequency (Hz)
d.c. (pA)

Figure S4. Experimental and simulated firing patterns of ND and RIB neurons.

A, For each neuron type, intracellularly recorded firing pattern from the mouse cortex (Experiment) and simulated activity (Simulation). Value of injected current is indicated below each trace. B, Plots of frequency versus injected current and histograms of minimal and maximal bursting frequencies for ND and RIB neurons as observed in the experiments and the simulations. Dashed line on the left of the traces indicates -60 mV. Experimental data are reproduced with permission from Lorincz, Gunner, Bao, Connelly, Isaac, Hughes and Crunelli (2015)[2].

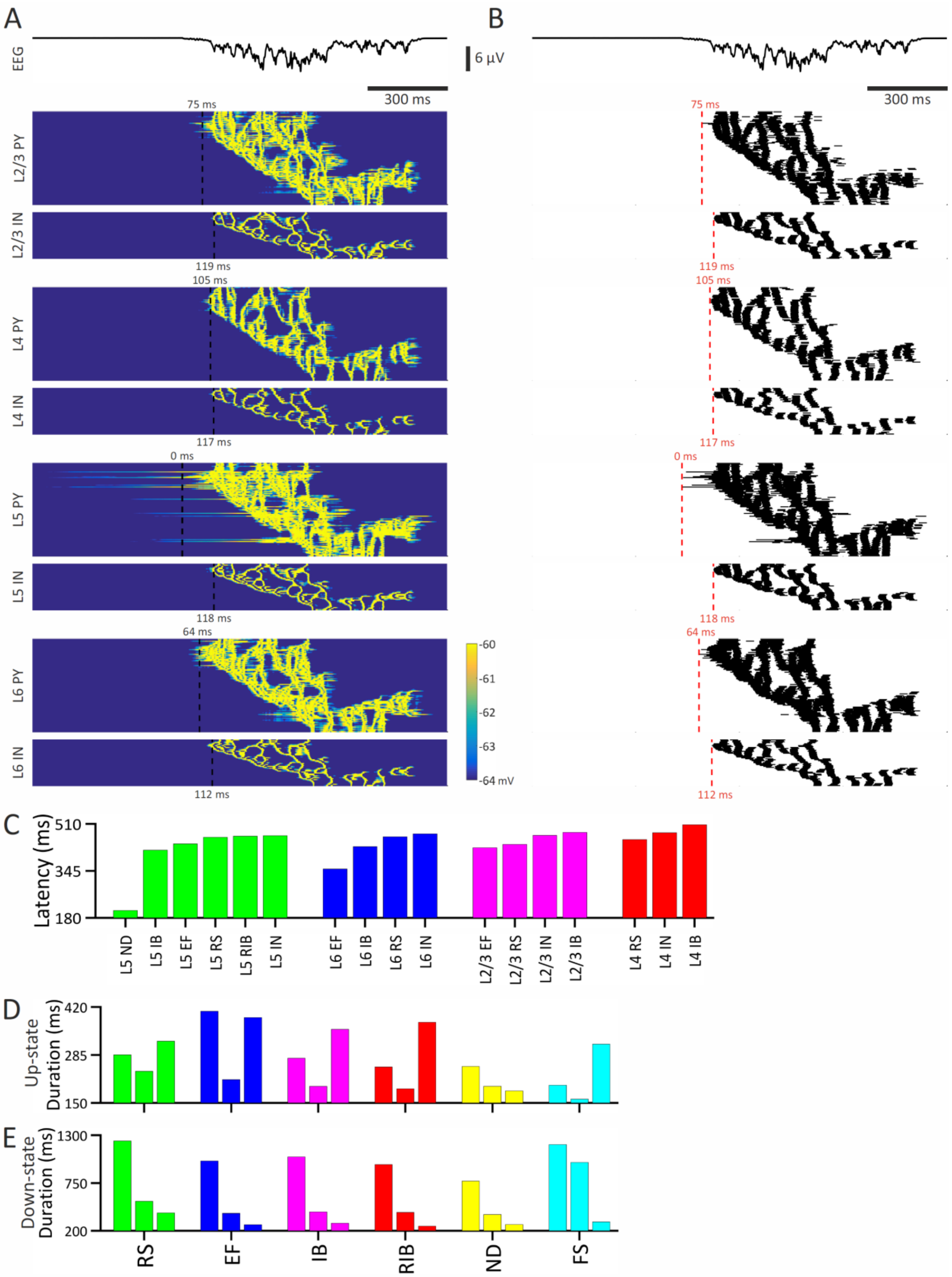
A
B
EEG
6 µV
300 ms
L2/3 PY
L2/3 IN
L4 PY
L4 IN
L5 PY
L5 IN
L6 PY
L6 IN
75 ms
119 ms
105 ms
117 ms
0 ms
118 ms
64 ms
112 ms
-60
-61
-62
-63
-64 mV
C
Latency (ms)
510
345
180
L5 ND
L5 IB
L5 EF
L5 RS
L5 RIB
L5 IN
L6 EF
L6 IB
L6 RS
L6 IN
L2/3 EF
L2/3 RS
L2/3 IN
L2/3 IB
L4 RS
L4 IN
L4 IB
D
Up-state Duration (ms)
420
285
150
E
Down-state Duration (ms)
1300
750
200
RS
EF
IB
RIB
ND
FS

Figure S5. Onset timing of firing in simulated Up-states in the isolated cortical network. A, EEG (top trace) and colour-coded membrane potential graphs of the indicated cortical neuronal populations during a cycle of the slow (< 1Hz) oscillation. B, EEG (top trace) and AP rastergrams corresponding to the colour-coded graphs of the neuronal populations indicated in A. Red dashed vertical line represents the first AP of the Up-state in each population. The latency (indicated in red below each rastergram) is measured relatively to the first AP of the cycle in a layer 5 pyramidal neurons (time zero). C, Mean onset latency histograms of the first AP for all cortical neurons grouped by their type measured with respect to the first AP of an Up-state. D and E, Up- and Down-state durations, respectively, averaged over a 300 sec-long simulation.

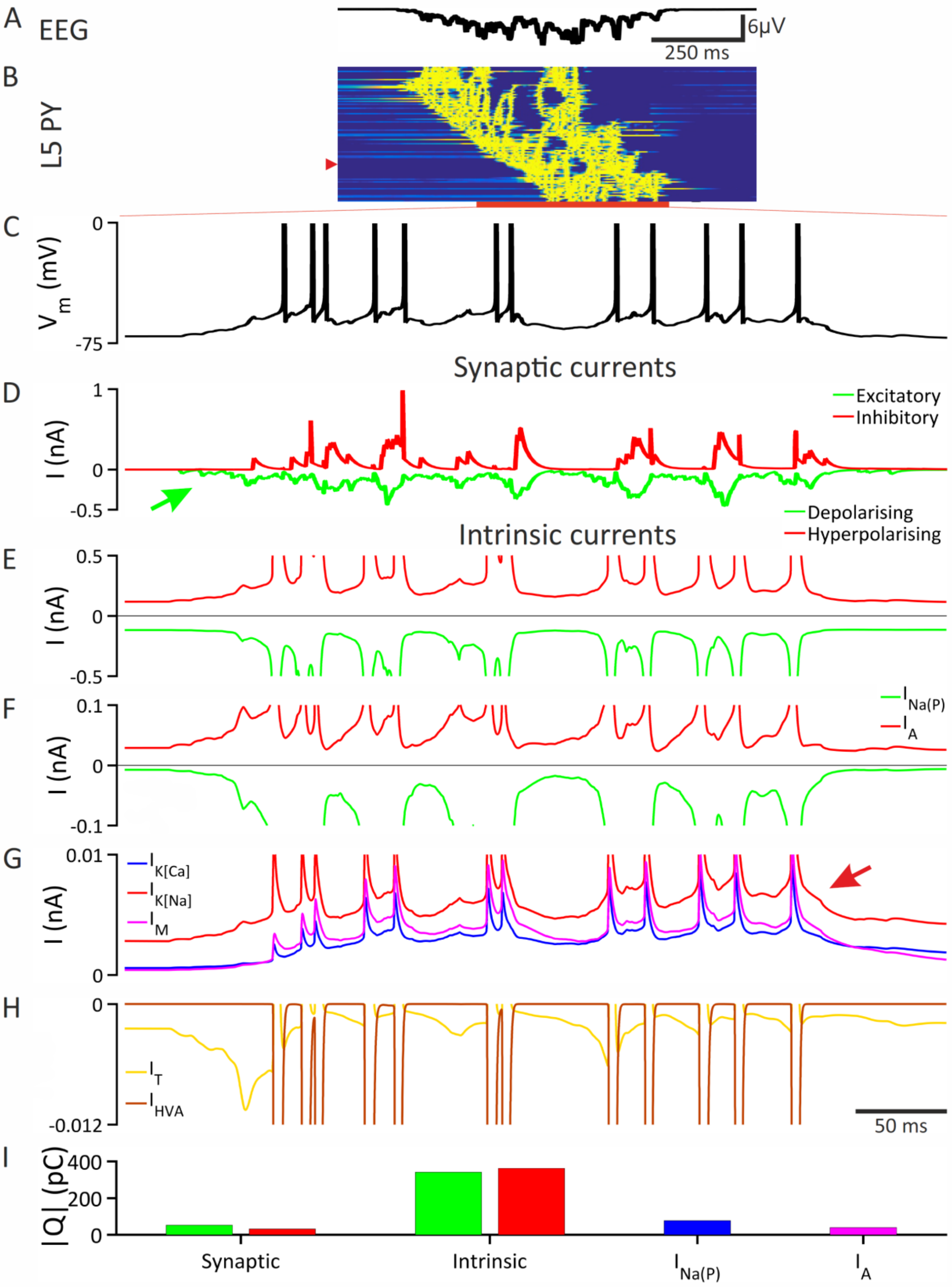
A EEG
6μV
250 ms
B L5 PY
C
0
-75
V_m (mV)
Synaptic currents
D
1
0
-0.5
I (nA)
Excitatory
Inhibitory
Depolarising
Hyperpolarising
Intrinsic currents
E
0.5
0
-0.5
I (nA)
F
0.1
0
-0.1
I (nA)
$I_{Na(P)}$
I_A
G
0.01
0
I (nA)
$I_{K[Ca]}$
$I_{K[Na]}$
I_M
H
0
-0.012
I_T
I_{HVA}
50 ms
I
|Q| (pC)
400
200
0
Synaptic
Intrinsic
$I_{Na(P)}$
I_A

Figure S6. Membrane currents involved in generating Up-states in the IB neuron model. A, Simulated EEG showing an UP-state of the slow (< 1Hz) oscillation. B, Colour-coded membrane potential graph of layer 5 pyramidal neurons (L5 PY) during the UP-state shown in A. C, Intracellularly recorded Up-state of the IB neuron marked by the red arrowhead in B. D, Total excitatory and inhibitory synaptic currents during the Up-state shown in C. The green arrow points to EPSPs initiating the Up-state. E, Total current of all intrinsic membrane channels during the Up-state shown in C. E-G, Specific intrinsic membrane currents as indicated in each panel during the Up-state shown in C ($I_{Na(P)}$: persistent Na^+ current; I_A: A current; $I_{K[Ca]}$: Ca^{2+}-activated K^+ current; $I_{K[Na]}$, Na^+-activated K^+ current; I_M: M current; I_T: T-type Ca^{2+} current; I_{HVA}: high-threshold Ca^{2+} current). Red arrow in G points to the accumulation of K^+ currents that contributes to the termination of the Up-state. Some currents are truncated for clarity. I, Plots of the indicated total currents during the Up-state shown in C.

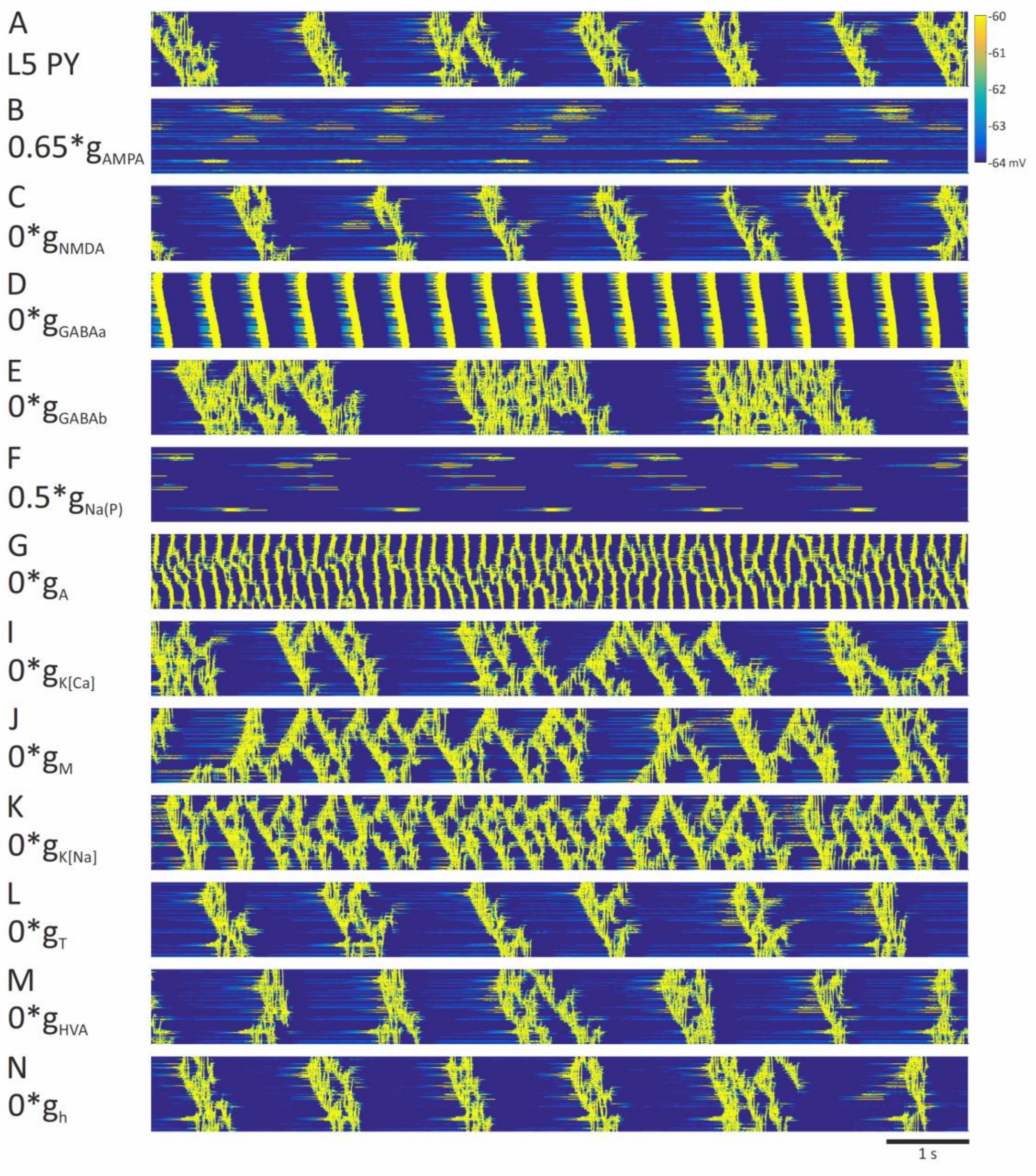

Figure S7. Role of various synaptic and intrinsic membrane currents in generating Up-states in the isolated neocortical network.

A, colour-coded membrane potential graphs of the pyramidal neuron population in layer 5 (L5 PY) during simulated slow (<1 Hz) oscillations in the isolated cortical network. B-N, As in A but for simulations obtained following the manipulation of the conductances (g) indicated to the left of each graph (g_{AMPA}: AMPA receptor conductance; g_{NMDA}: NMDA receptor conductance; g_{GABAa}: GABA-A receptor conductance; g_{GABAb}: GABA-B receptor conductance; $g_{Na(P)}$: persistent Na^+ current conductance; g_A: A current conducatnce; $g_{K(Ca)}$: Ca^{2+}-activated K^+ conductance; g_M: M current conductance; $g_{K(Na)}$: Na^+-activated K^+ conductance; g_T: T-type Ca^{2+} conductance; g_{HVA}: high-voltage Ca^{2+} conductance; g_h: hyperpolarization-activated cyclic nucleotide-gated conductance.

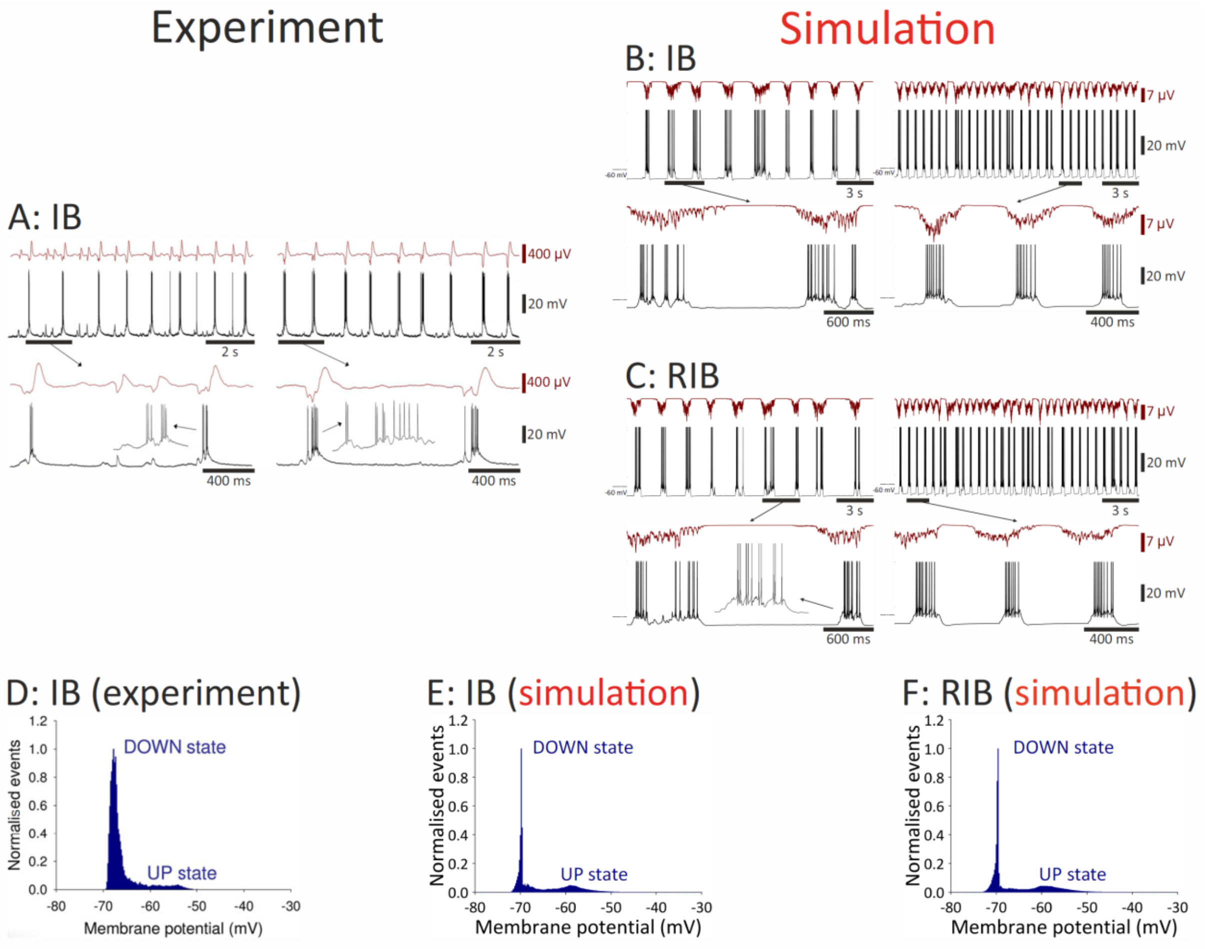

Figure S8. Experimental and simulated membrane potential dynamics of IB and RIB neurons during slow (<1 Hz) oscillations in the isolated cortical network.

A,B, Local field potential (top trace) and membrane potential dynamics of an IB neuron recorded *in vitro* (Experiment) and in the isolated cortical network (Simulation) during slow (<1 Hz) oscillations. C, Local field potential (top trace) and membrane potential dynamics of an RIB neuron during simulated slow (<1 Hz) oscillations (Simulation). In A, B and C, the left-hand traces in each pair were recorded during an early appearance of the slow (<1 Hz) oscillation while the right-hand traces show the oscillation at a later stage. D, E, F, Histograms of the normalized membrane potential distribution with the typical peaks of the Up- and Down-state for the experimental and simulated data of an IB neuron and for the simulated results of an RIB neuron. Experimental data are reproduced with permission from Lorincz, Gunner, Bao, Connelly, Isaac, Hughes and Crunelli (2015)[2].

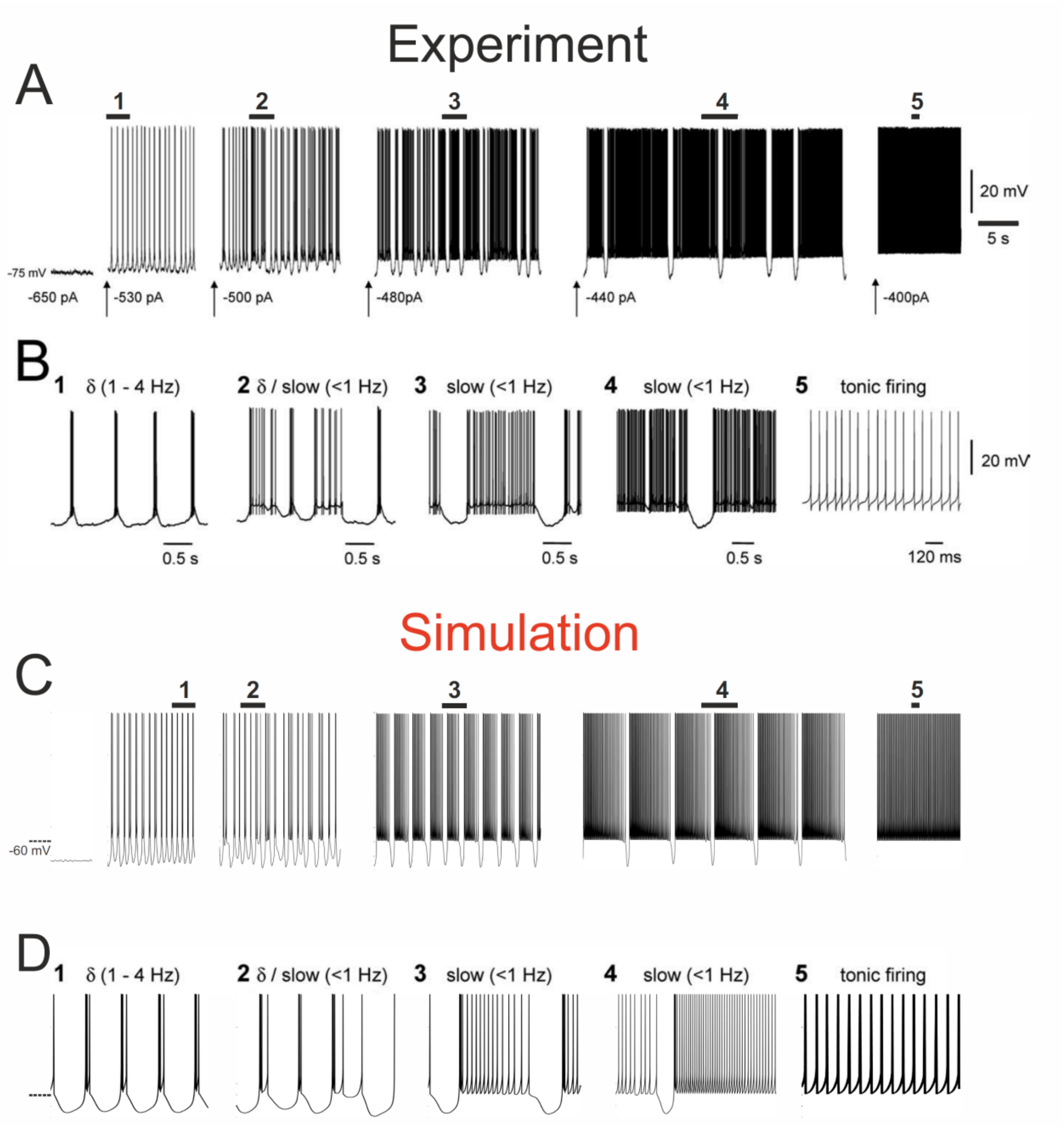

Figure S9. Intrinsic activity of NRT neurons.

A, Delta waves, slow (< 1Hz) oscillations and tonic firing of a cat NRT neuron recorded *in vitro*. B, Enlargement of the traces marked by numbers in A. C, Simulated delta waves, slow (< 1Hz) oscillations and tonic firing of an NRT neuron. D, Enlargement of the trace marked by numbers in C. Experimental data are reproduced with permission from Blethyn, Hughes, Tóth, Cope and Crunelli (2006)[3].

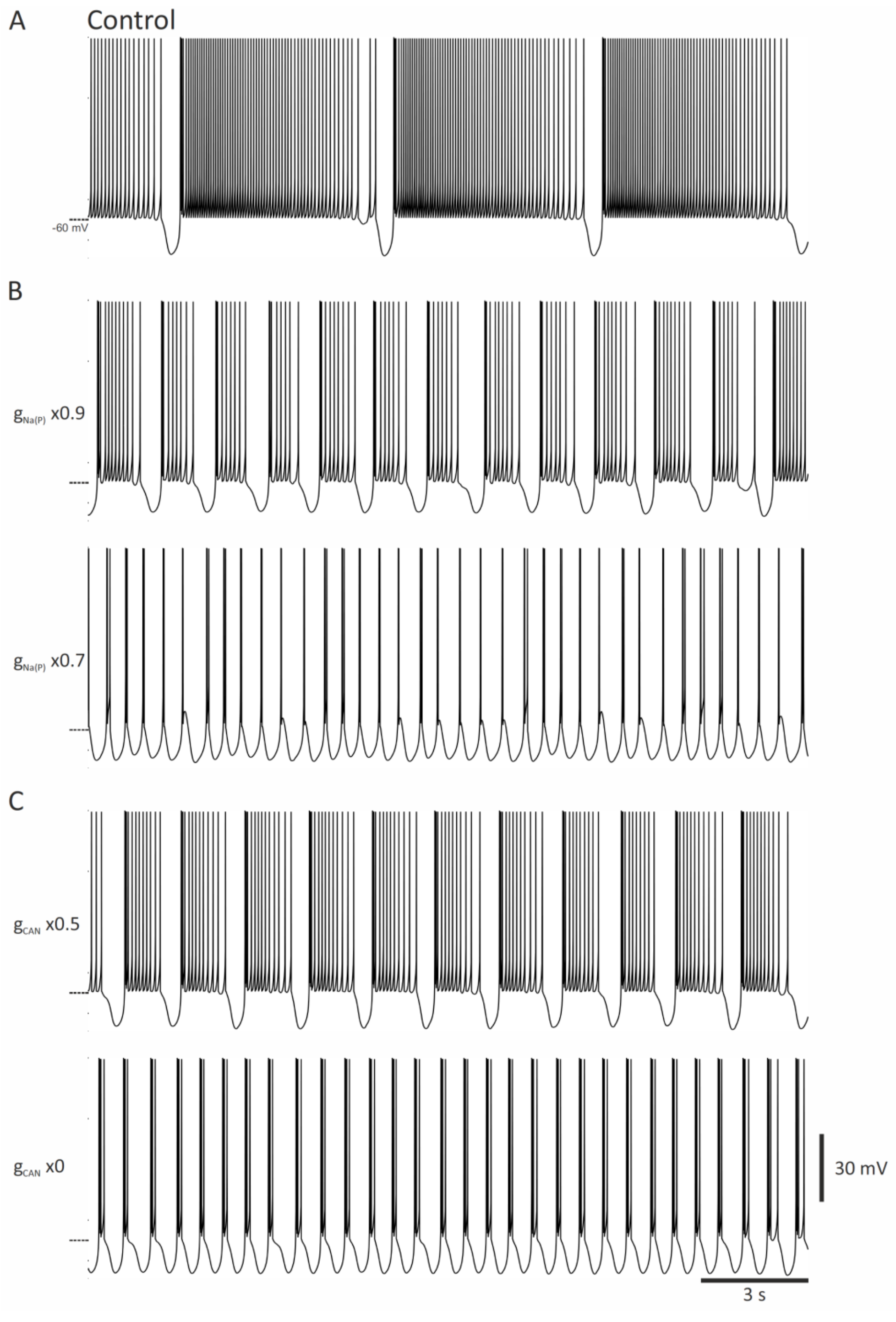

Figure S10. Role of $I_{Na(P)}$ and I_{CAN} in the slow (< 1Hz) oscillation of NRT_{FO} neurons.

A, Simulated slow (< 1Hz) oscillations of an NRT_{FO} neuron under control condition. B, Reducing the conductance of the persistent Na^+ current ($g_{Na(P)}$) gradually reduces the Up-state duration of the slow (< 1Hz) oscillation, eventually leading to the expression of delta waves (bottom trace). C, Reducing and blocking the conductance of the non-selective cation current (g_{CAN}) increases the frequency of the simulated slow (< 1Hz) oscillation and eventually abolishes it, leading to delta waves (bottom trace).

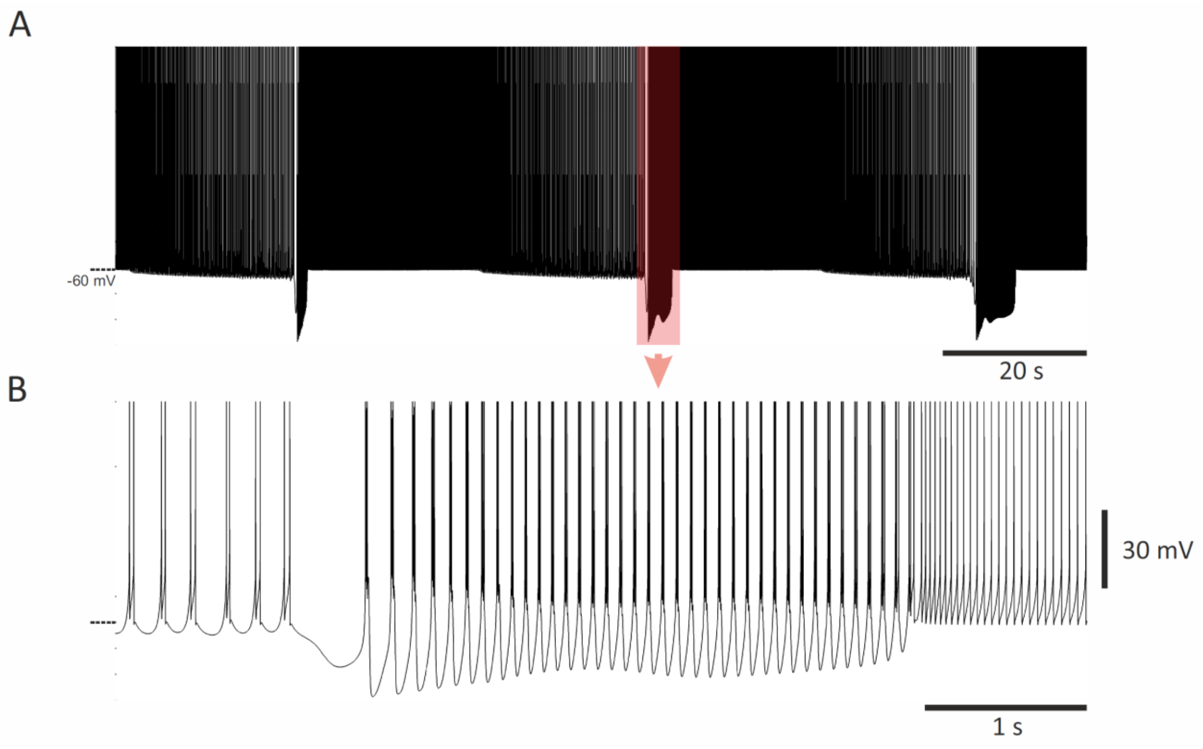

Figure S11. Spindle waves in isolated NRT_{FO} neurons.

A, The trace shows 3 fast (~9-12 Hz) spindle waves that were generated spontaneously in NRT_{FO} neurons when they are isolated from TC_{FO} neurons. B, Enlargement of the spindle wave highlight in A.

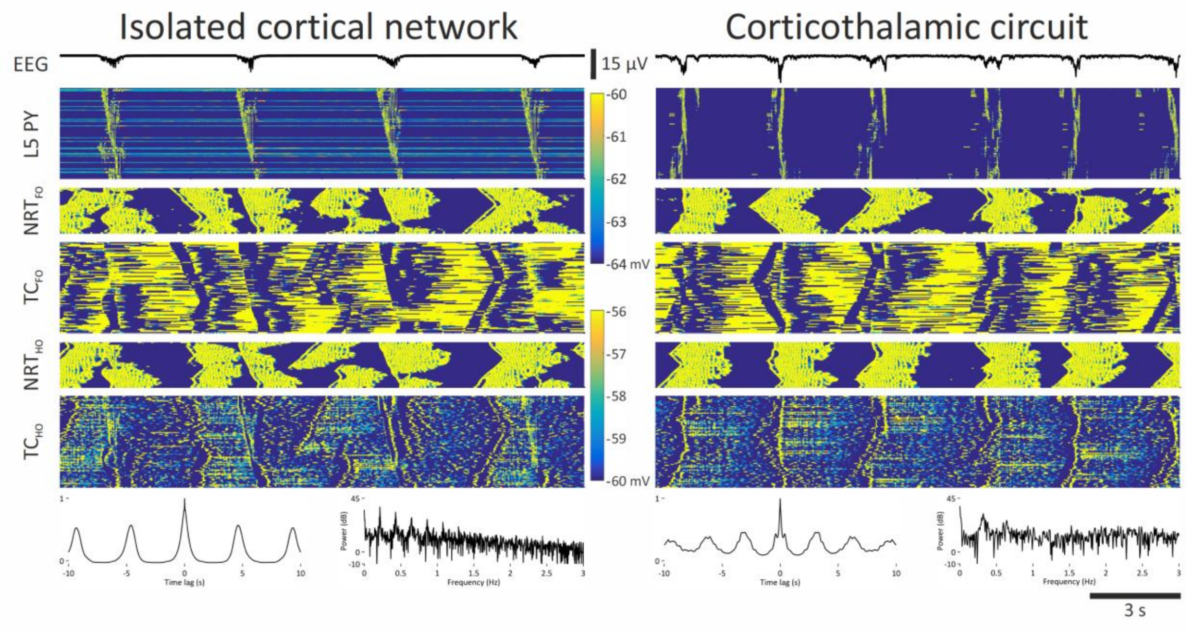

Figure S12. The thalamic input increases the frequency of the slow (<1 Hz) oscillation. Left panel: simultaneous EEG (top trace) and colour-coded membrane potential graphs of the indicated neuronal populations during slow (<1 Hz) oscillations simulated in the isolated cortical model. EEG autocorrelograms (left) and power graphs (right) are shown at the bottom. Right panel: as on the left but with an active thalamocortical input. Note the increased regularity and frequency of the slow (< 1Hz) oscillation in B compared to that shown in A. L5 PY: pyramidal neurons in cortical layer 5; NRT_{FO}: first order NRT neurons; TC_{FO}: first order TC neurons; NRT_{HO}: higher order NRT neurons; TC_{HO}: higher order TC neurons.

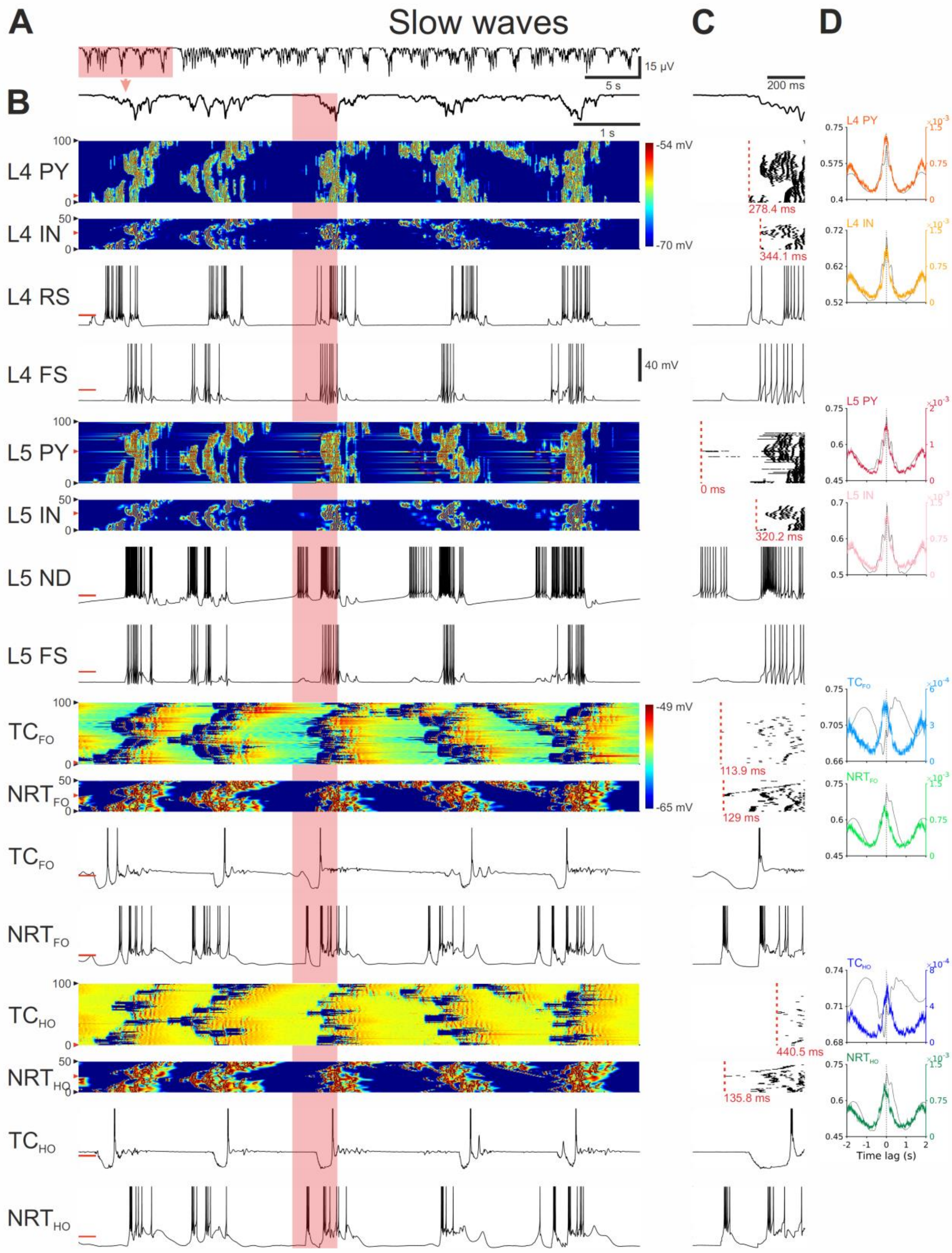

A
Slow waves
C
D
15 µV
5 s
B
1 s
200 ms
L4 PY
L4 IN
L4 RS
L4 FS
L5 PY
L5 IN
L5 ND
L5 FS
TC FO
NRT FO
TC FO
NRT FO
TC HO
NRT HO
TC HO
NRT HO
-54 mV
-70 mV
40 mV
-49 mV
-65 mV
278.4 ms
344.1 ms
0 ms
320.2 ms
113.9 ms
129 ms
440.5 ms
135.8 ms
Time lag (s)

Figure S13. Slow (<1Hz) oscillations in the full corticothalamic model start in L5 when ND neurons are depolarized.

A, EEG showing the rhythmic pattern of slow (< 1Hz) oscillations. B, EEG (top trace) and colour-coded membrane potential plots of the indicated cortical and thalamic neuronal populations during the 5 cycles of the slow (< 1Hz) oscillation highlighted in A (note the two separate colour-scales for the cortical and thalamic neurons). Below are the corresponding membrane potential waveforms of the two neurons indicated by the red arrow on the left of the corresponding colour-coded plots. C, EEG (top trace) and AP rastergrams of the firing in each neuronal population for the slow (< 1Hz) oscillation cycle highlighted in B. Red dashed vertical line represents the first AP of the Up-state in each population. The latency (indicated in red below each rastergram) is measured relatively to the first AP of the cycle in the L5 neuron that fires first (time zero). Below the rastergrams are the corresponding membrane potential waveforms of that cycle for the indicated neuron. D, Cross-correlations of EEG and APs for the indicated neuronal populations, calculated over a 485 sec-long simulation. Shaded regions are 95% confidence intervals. Dashed vertical line indicates the zero lag. L4 PY: pyramidal neurons in cortical layer 4; L4 IN: interneurons in cortical layer 4; L4 IB: IB neuron in cortical layer 4; L4 FS: FS neuron in cortical layer 4; L5 PY: pyramidal neurons in cortical layer 5; L5 IN: interneurons in cortical layer 5; L5 RS: RS neuron in cortical layer 5; L5 FS: FS neuron in cortical layer 5; TC_{FO}: first order TC neurons; TC_{HO}: higher order TC neurons; NRT_{FO}: first order NRT neurons; NRT_{HO}: higher order NRT neurons.

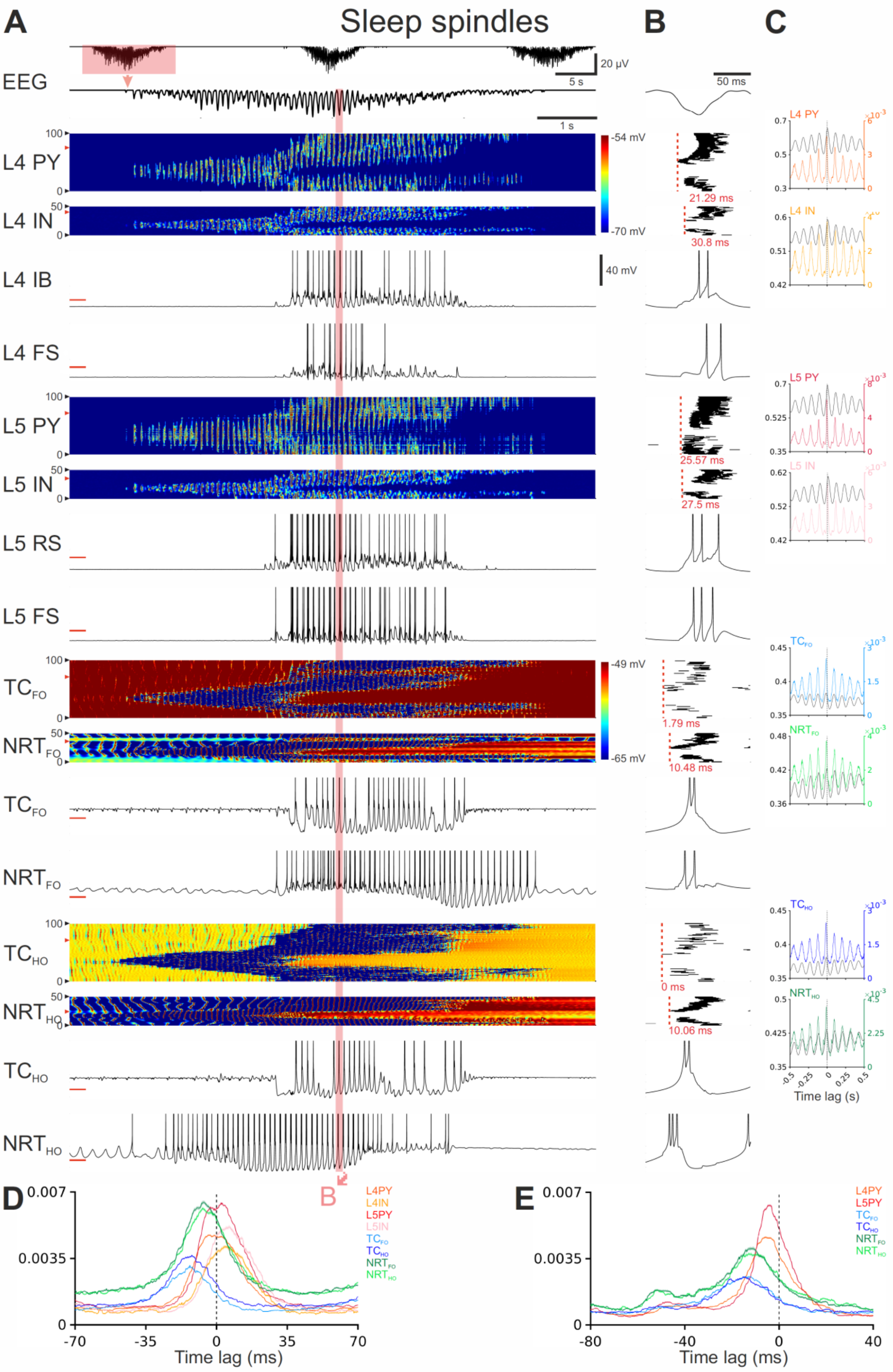

A
Sleep spindles
B
C
EEG
20 µV
5 s
1 s
50 ms
L4 PY
L4 IN
L4 IB
L4 FS
L5 PY
L5 IN
L5 RS
L5 FS
TCFO
NRTFO
TCHO
NRTHO
-54 mV
-70 mV
40 mV
-49 mV
-65 mV
21.29 ms
30.8 ms
25.57 ms
27.5 ms
1.79 ms
10.48 ms
0 ms
10.06 ms
Time lag (s)
D
E
Time lag (ms)
L4PY
L4IN
L5PY
L5IN

Figure S14. Sleep spindles in the full corticothalamic model.

A, Top trace: EEG showing the rhythmic pattern of sleep spindles. The highlighted spindle wave is enlarged below. Lower traces: colour-coded membrane potential plots of the indicated cortical and thalamic neuron populations (note the two separate colour-coded scales for the cortex and the thalamus). Below are the corresponding membrane potential waveforms of the two neurons indicated by the red arrow on the left of the corresponding colour-coded plots. B, EEG (top trace), AP rastergrams of the firing of the first AP in each neuronal population for the sleep spindle cycle highlighted in B. Red dashed vertical line represents the first AP of the Up-state in each population. The latency (indicated below each rastergram) is measured relatively to the first AP of the cycle in a TC_{HO} neuron (time zero). Below the rastergrams are the corresponding membrane potential waveforms of that cycle for the indicated neuron. C, Cross-correlations of EEG and APs for the indicated neuronal populations, calculated over a 485 sec-long simulation. Shaded regions are 95% confidence intervals. Dashed vertical line indicates the zero lag. D, AP distribution with respect to the EEG for all APs of the indicated neuronal populations. Shaded regions are 95% confidence intervals. Dashed vertical line indicates the zero lag. E, Distribution of the first AP in a spindle cycle with respect to the EEG for all APs of the indicated neuronal populations. Shaded regions are 95% confidence intervals. Dashed vertical line indicates the zero lag. L4 PY: pyramidal neurons in cortical layer 4; L4 IN: interneurons in cortical layer 4; L4 IB: IB neuron in cortical layer 4; L4 FS: FS neuron in cortical layer 4; L5 PY: pyramidal neurons in cortical layer 5; L5 IN: interneurons in cortical layer 5; L5 RS: RS neuron in cortical layer 5; L5 FS: FS neuron in cortical layer 5; TC_{FO}: first order TC neurons; TC_{HO}: higher order TC neurons; NRT_{FO}: first order NRT neurons; NRT_{HO}: higher order NRT neurons.

Supplementary Tables

Table S1. Network connectivity parameters.

Grp	Src	Target	Type	P (%)	Amp (mV), weight	Mini (mV), weight	Del (ms)	RT (ms)	τ_D (ms)
1.1	$L2/3_E$	$L2/3_E$	AMPA	13	0.8, 1.05	0.17, 0.23	3	4.4	20
			NMDA	13	0.06*, 1.05	-	3	13.2	135
1.2	$L2/3_E$	$L2/3_I$	AMPA	13	0.8, 1.05	0.17, 0.17	1.5	4.4	22
			NMDA	13	0.06*, 1.05	-	1.5	13.2	90
1.3	$L2/3_I$	$L2/3_E$	$GABA_A$	13	1-1.5, 2.25	0.083, 0.083	1.5	3.5	22
			$GABA_B$	13	4.25**, 2.25	-	1.5	42.5	90
2.1	$L2/3_E$	$L4_E$	AMPA	3	0.8, 1.05	0.17, 0.23	2.5	4.4	20
			NMDA	3	0.06*, 1.05	-	2.5	13.2	135
2.2	$L2/3_E$	$L4_I$	$GABA_A$	3	1-1.5, 2.25	0.17, 0.17	2.5	3.5	22
			$GABA_B$	3	4.25**, 2.25	-	2.5	42.5	90
3.1	$L2/3_E$	$L5_E$	AMPA	20	0.8, 1.05	0.17, 0.23	2.75	4.4	20
			NMDA	20	0.06*, 1.05	-	2.75	13.2	135
3.2	$L2/3_E$	$L5_I$	$GABA_A$	20	1-1.5, 2.25	0.17, 0.17	2.75	3.5	22
			$GABA_B$	20	4.25**, 2.25	-	2.75	42.5	90
4.1	$L4_E$	$L4_E$	AMPA	15	0.8 1.05	0.17, 0.23	3	4.4	20
			NMDA	15	0.06* 1.05	-	3	13.2	135
4.2	$L4_E$	$L4_I$	AMPA	15	0.8 1.05	0.17, 0.17	1.5	4.4	20
			NMDA	15	0.06* 1.05	-	1.5	13.2	135
4.3	$L4_I$	$L4_E$	$GABA_A$	15	1-1.5, 2.25	0.083, 0.083	1.5	3.5	22
			$GABA_B$	15	4.25**, 2.25	-	1.5	42.5	90
5.1	$L4_E$	$L2/3_E$	AMPA	25	0.8, 1.05	0.17, 0.23	2.5	4.4	20
			NMDA	25	0.06*, 1.05	-	2.5	13.2	135
5.2	$L4_E$	$L2/3_I$	$GABA_A$	25	1-1.5, 2.25	0.17, 0.17	2.5	3.5	22
			$GABA_B$	25	4.25**, 2.25	-	2.5	42.5	90
6.1	$L4_E$	$L5_E$	AMPA	9	0.8, 1.05	0.17, 0.23	2.5	4.4	20
			NMDA	9	0.06*, 1.05	-	2.5	13.2	135
6.2	$L4_E$	$L5_I$	$GABA_A$	9	1-1.5, 2.25	0.17, 0.17	2.5	3.5	22
			$GABA_B$	9	4.25**, 2.25	-	2.5	42.5	90
7.1	$L4_E$	$L6_E$	AMPA	9	0.8, 1.05	0.17, 0.23	2.75	4.4	20
			NMDA	9	0.06*, 1.05	-	2.75	13.2	135
7.2	$L4_E$	$L6_I$	$GABA_A$	9	1-1.5, 2.25	0.17, 0.17	2.75	3.5	22
			$GABA_B$	9	4.25**, 2.25	-	2.75	42.5	90
8.1	$L5_E$	$L5_E$	AMPA	10	0.8, 1.05	0.17, 0.23	3	4.4	20
			NMDA	10	0.06*, 1.05	-	3	13.2	135
8.2	$L5_E$	$L5_I$	AMPA	10	0.8, 1.05	0.17, 0.17	1.5	4.4	20
			NMDA	10	0.06*, 1.05	-	1.5	13.2	135
8.3	$L5_I$	$L5_E$	$GABA_A$	10	1-1.5, 2.25	0.083, 0.083	1.5	3.5	22
			$GABA_B$	10	4.25**, 2.25	-	1.5	42.5	90
9.1	$L5_E$	$L2/3_E$	AMPA	9	0.8, 1.05	0.17, 0.23	2.75	4.4	20
			NMDA	9	0.06*, 1.05	-	2.75	13.2	135
9.2	$L5_E$	$L2/3_I$	$GABA_A$	9	1-1.5, 2.25	0.17, 0.17	2.75	3.5	22
			$GABA_B$	9	4.25**, 2.25	-	2.75	42.5	90

10.1	$L5_E$	$L4_E$	AMPA	3	0.8, 1.05	0.17, 0.23	2.5	4.4	20
			NMDA	3	0.06*, 1.05	-	2.5	13.2	135
10.2	$L5_E$	$L4_I$	$GABA_A$	3	1-1.5, 2.25	0.17, 0.17	2.5	3.5	22
			$GABA_B$	3	4.25**, 2.25	-	2.5	42.5	90
11.1	$L5_E$	$L6_E$	AMPA	15	0.8, 1.05	0.17, 0.23	2.5	4.4	20
			NMDA	15	0.06*, 1.05	-	2.5	13.2	135
11.2	$L5_E$	$L6_I$	$GABA_A$	15	1-1.5, 2.25	0.17, 0.17	2.5	3.5	22
			$GABA_B$	15	4.25**, 2.25	-	2.5	42.5	90
12.1	$L6_E$	$L6_E$	AMPA	10	0.8, 1.05	0.17, 0.23	3	4.4	20
			NMDA	10	0.06*, 1.05	-	3	13.2	135
12.2	$L6_E$	$L6_I$	AMPA	10	0.8, 1.05	0.17, 0.17	1.5	4.4	20
			NMDA	10	0.06*, 1.05	-	1.5	13.2	135
12.3	$L6_I$	$L6_E$	$GABA_A$	10	1-1.5, 2.25	0.083, 0.083	1.5	3.5	22
			$GABA_B$	10	4.25**, 2.25	-	1.5	42.5	90
13.1	$L6_E$	$L4_E$	AMPA	15	0.8, 1.05	0.17, 0.23	3.5	4.4	20
			NMDA	15	0.06*, 1.05	-	3.5	13.2	135
13.2	$L6_E$	$L4_I$	$GABA_A$	15	1-1.5, 2.25	0.17, 0.17	3.5	3.5	22
			$GABA_B$	15	4.25**, 2.25	-	3.5	42.5	90
14.1	$L6_E$	$L5_E$	AMPA	3	0.8, 1.05	0.17, 0.23	3.25	4.4	20
			NMDA	3	0.06*, 1.05	-	3.25	13.2	135
14.2	$L6_E$	$L5_I$	$GABA_A$	3	1-1.5, 2.25	0.17, 0.17	3.25	3.5	22
			$GABA_B$	3	4.25**, 2.25	-	3.25	42.5	90
15.1	NRT_{FO}	NRT_{FO}	$GABA_A$	10	0.10, 0.028	0.25, 0.1	1	12	38
15.2	NRT_{HO}	NRT_{HO}	$GABA_A$	10	0.10, 0.028	0.25, 0.1	1	12	38
16.1	NRT_{FO}	TC_{FO}	$GABA_A$	7.5	0.4, 0.6	0.5, 0.5	2.5	2.4	30
			$GABA_B$	7.5	0.8**, 0.6	-	2.5	90	70
16.2	NRT_{HO}	TC_{HO}	$GABA_A$	7.5	0.4, 0.6	0.5, 0.5	2.5	2.4	30
			$GABA_B$	7.5	0.8**, 0.6	-	2.5	90	70
17.1	NRT_{FO}	TC_{HO}	$GABA_A$	2.5	0.4, 0.6	0.5, 0.5	2.5	2.4	30
			$GABA_B$	2.5	0.8**, 0.6	-	2.5	90	70
17.2	NRT_{HO}	TC_{FO}	$GABA_A$	2.5	0.4, 0.6	0.5, 0.5	2.5	2.4	30
			$GABA_B$	2.5	0.8**, 0.6	-	2.5	90	70
18.1	TC_{FO}	NRT_{FO}	AMPA	3.75	4, 0.44	0.5, 0.052	1	0.6	16
			NMDA	3.75	0.1*, 0.44	-	1	13.5	75
18.2	TC_{HO}	NRT_{HO}	AMPA	3.75	4, 0.44	0.5, 0.052	1	0.6	16
			NMDA	3.75	0.1*, 0.44	-	1	13.5	75
19.1	TC_{FO}	NRT_{HO}	AMPA	1.25	4, 0.44	0.5, 0.052	1	0.6	16
			NMDA	1.25	0.1*, 0.44	-	1	13.5	75
19.2	TC_{HO}	NRT_{FO}	AMPA	1.25	4, 0.44	0.5, 0.052	1	0.6	16
			NMDA	1.25	0.1*, 0.44	-	1	13.5	75
20.1	TC_{FO}	$L4_E$	AMPA	16	5.5, 8.4	0.17, 0.17	4	4.3	20.4
			NMDA	16	0.4*, 8.4	-	4	13.6	129
20.2	TC_{FO}	$L4_I$	AMPA	4	7, 8.4	0.17, 0.17	4	3.9	18.8
			NMDA	4	0.5*, 8.4	-	4	12.6	137
21.1	TC_{FO}	$L5_E$	AMPA	16	0.5, 0.7	0.17, 0.17	4	5.2	19.4
			NMDA	16	0.03*, 0.7	-	4	13	138
21.2	TC_{FO}	$L5_I$	AMPA	4	0.65, 0.7	0.17, 0.17	4	4.2	20.1

			NMDA	4	0.04*, 0.7	-	4	13	137
22.1	TC_{HO}	$L2/3_E$	AMPA	32	2.4, 3.5	0.17, 0.17	6	4.3	20.7
			NMDA	32	0.2*, 3.5	-	6	13.2	142
22.2	TC_{HO}	$L2/3_I$	AMPA	8	3.1, 3.5	0.17, 0.17	6	4.3	19.3
			NMDA	8	0.2*, 3.5	-	6	12.9	139
23.1	TC_{HO}	$L4_E$	AMPA	32	0.5, 0.7	0.17, 0.17	6	5.1	20.2
			NMDA	32	0.03*, 0.7	-	6	13.1	132
23.2	TC_{HO}	$L4_I$	AMPA	8	0.65, 0.7	0.17, 0.17	6	4.2	20.1
			NMDA	8	0.04*, 0.7	-	6	13.7	122
24.1	TC_{HO}	$L5_E$	AMPA	32	3.3, 4.9	0.17, 0.17	6	4.5	20.8
			NMDA	32	0.25*, 4.9	-	6	14.7	131
24.2	TC_{HO}	$L5_I$	AMPA	8	4.3, 4.9	0.17, 0.17	6	4	19.1
			NMDA	8	0.3*, 4.9	-	6	12.3	139
25.1	TC_{HO}	$L6_E$	AMPA	32	2.4, 3.5	0.17, 0.17	6	4.5	20.2
			NMDA	32	0.2*, 3.5	-	6	13.5	131
25.2	TC_{HO}	$L6_I$	AMPA	8	3.1, 3.5	0.17, 0.17	6	4.3	19.3
			NMDA	8	0.2*, 3.5	-	6	12.9	139
26	$L5_E$	TC_{HO}	AMPA	10	0.025†, 0.05	0.5, 0.5	4	1	24.1
			NMDA	10	0.0035‡, 0.05	-	4	20.2	83.3
27	$L6_E$	NRT_{FO}	AMPA	10	1.3, 0.132	0.5, 0.52	8	0.8	16.4
			NMDA	10	0.3*, 0.132	-	8	15	80
28	$L6_E$	TC_{FO}	AMPA	10	0.5†, 0.09	0.5, 0.5	4	0.7	22.4
			NMDA	10	0.014‡, 0.09	-	4	22	74.4
29	$L6_E$	NRT_{HO}	AMPA	10	1.3, 0.132	0.5, 0.52	8	0.8	16.4
			NMDA	10	0.3*, 0.132	-	8	15	80
30	$L6_E$	TC_{HO}	AMPA	10	0.5†, 0.09	0.5, 0.5	4	0.7	22.4
			NMDA	10	0.014‡, 0.09	-	4	22	74.4

* Estimated at the resting V_M when extracellular Mg^{2+} concentration is set to 0.1 mM.

** Estimated at the resting V_M in response to a train of 10 presynaptic APs at 100 Hz frequency.

† Estimated at V_M = -80 mV to avoid activation of T-type Ca^{2+} channels.

‡ Estimated at V_M = -80 mV and with extracellular Mg^{2+} concentration set to 0.1 mM.

Abbreviations: E, excitatory cell; I, inhibitory; Grp, projection group; Src, source; P, proportion of connected cells in the target structure; Amp, amplitude; Mini, amplitude of a miniature or spontaneous PSP; Del, delay or latency from an AP in the presynaptic cell (when -10 mV threshold is passed) to the initiation of a PSP; RT, rise time; τ_D, the time it takes for a PSP amplitude to decay to Amp/e, where e is the Euler number.

Table S2. Derivation of network connectivity parameters.

Grp	Type	P	Weight	Delay	Shape
1-14	AMPA, NMDA	[4,5,6,7,8,9,10,11,12,13,14,15,16,17,18,19,20,21,22,23].		[18,21,24,25,26,27].	[28,29,30,31,32].
	$GABA_A$	[4,5,6,7,8,9,10,11,12,13,14,15,16,17,18,19,20,21,22,23].		[18,21,24,25,26,27,33].	[34,35,36,37,38,39,40].
	$GABA_B$	[4,5,6,7,8,9,10,11,12,13,14,15,16,17,18,19,20,41,42].		[18,21,24,25,26,27,33].	[40].
15	$GABA_A$	[43,44,45].	[45,46,47,48,49].	[46,47,48,49,50,51,52,53,54,55,56,57,58].	
16-17	$GABA_A$	[43,44,45,59].	[47,50,51,60,61,62,63].	[64,65].	[47,50,51].
	$GABA_B$	[43,44,45,59].	[47,50,51].	[64,65].	[46,61].
18-19	AMPA, NMDA		[61,62,65,66].		
20-25	AMPA, NMDA, $GABA_A$, $GABA_B$		[59,67,68,69,70,71,72,73,74,75,76,77,78,79,80,81,82,83,84,85,86,87,88,89,90].		
26-30	AMPA NMDA		[59,63,91,92,93,94,95,96,97,98,99,100,101,102,103,104,105,106].		

Table S3. The size of active conductances in TC cells

Cell	$\bar{g}_{Na}$	$\bar{g}_{K(DR)}$	$\bar{g}_{A1}$	$\bar{g}_{A2}$	$\bar{g}_{K1}$	$\bar{g}_{K2}$	$\bar{g}_{CAN}$	$\bar{g}_{h}$	$\bar{g}_{Na(P)}$	$\bar{g}_{T}$	$\bar{P}_{HVA}$
TC_{FO}	70	70	0.242	0.16045	0.014	0.2	0.075	8.5	0.2015	2.1	0.135
TC_{HO}	70	70	0.242	0.16045	0.014	0.2	0.075	8.5	0.2015	4.2	0.135

The maximum conductances ($\bar{g}$) are expressed in μS/cm^2, whereas the maximum membrane permeability to Ca^{2+} ($\bar{P}$) is expressed in μm/s.

Table S4. The size of active conductances in NRT cells.

Cell	$\bar{g}_{Na}$	$\bar{g}_{K(DR)}$	$\bar{g}_{AHP1}$	$\bar{g}_{AHP2}$	$\bar{g}_{K[Na]}$	$\bar{g}_{CAN}$	$\bar{g}_{h}$	$\bar{g}_{Na(P)}$	$\bar{g}_{T}$	$\bar{g}_{HVA}$
NRT	50	50	0.003-0.3	0.0006-0.06	0.0002	0.0475	0.0043	0.01612	1.4	0.2

The maximum conductances ($\bar{g}$) are expressed in μS/cm^2.

Table S5. The size of cortical axosomatic and dendritic active conductances

Cell	$\bar{g}_{Na}$	$\bar{g}_{K(DR)}$	$\bar{g}_{A}$	$\bar{g}_{M}$	$\bar{g}_{fAHP}$	$\bar{g}_{sAHP}$	$\bar{g}_{h}$	$\bar{g}_{Na(P)}$	$\bar{g}_{K[Na]}$	$\bar{P}_{T}$	$\bar{g}_{HVA}$
RS	3000	-	-	-	-	-	-	0.077	0.07	-	-
	1.5	216	1.48	0.01	0.001	-	0.02	0.077	0.07	0.1	0.001
EF	3000	-	-	-	-	-	-	0.077	0.07	-	-
	1.5	216	1.48	0.01	0.001	-	0.02	0.077	0.07	0.1	0.001
IB	3000	-	-	-	-	-	-	0.077	0.07	-	-
	1.5	216	1.48	0.01	0.001	-	0.02	0.077	0.07	1	0.01
RIB	3000	-	-	-	-	-	-	0.077	0.07	-	-
	1.5	216	1.48	0.01	0.001	-	0.02	0.077	0.07	1	0.01
SIB	3000	-	-	-	-	-	-	0.077	0.07	-	-
	1.5	216	1.48	0.01	0.001	-	0.02	0.077	0.07	1	0.016
ND	3000	-	-	-	-	-	-	0.077	0.07	-	-
	1.5	216	1.48	0.01	0.001	0.03	0.02	0.077	0.07	1	0.016
FS	3000	-	-	-	-	-	-	-	0.07	-	-
	1.5	216	1.48	0.01	0.001	-	-	-	0.07	-	0.001

The maximum conductances ($\bar{g}$) are expressed in mS/cm^2, whereas the maximum membrane permeability to Ca^{2+} ($\bar{P}$) is expressed in µm/s. The top and bottom values for each neuron type correspond to axosomatic and dendritic compartments, respectively.

Table S6. Passive properties of model cells.

Cell	V_R (mV)	R_I (MΩ)	τ (ms)	G_{KL} (µS/cm^2)	E_{KL} (mV)	G_{NaL} (µS/cm^2)	E_{NaL} (mV)	C_m (µf/cm^2)	L (µm)	d (µm)
TC_{FO}	-65	160	15.5*	47	-90	9.1	10	0.88	90	60
TC_{HO}	-65	130	19.2*	51.2	-90	9.1	10	0.88	90	60
NRT	-65	160	13.7	89	-90	23.3	10	0.88	63	42
RS	-71.86	233	15	29.3	-90	7.8	10	0.75	-	-
EF	-65	>300	30	15	-90	7.8	10	0.75	-	-
IB	-71.67	227	14.8	29.3	-90	7.8	10	0.75	-	-
RIB	-71.67	222	14.6	29.3	-90	7.8	10	0.75	-	-
SIB	-71.65	216	14.8	29.3	-90	7.8	10	0.75	-	-
ND	-67.5	>250	25	29.3	-90	7.8	10	0.75	-	-
FS	-73	179	15.1	29.3	-90	7.8	10	0.75	-	-

* Estimated at V_M = -80 mV to avoid activation of T-type Ca^{2+} channels.

The apparent input resistance (R_I) was estimated by injecting a hyperpolarising 20 pA current at V_M = -60 mV. Abbreviations: V_R, resting membrane potential; R_I, apparent input resistance; τ, passive membrane time constant; E_{KL}, K^+ leak current reversal potential; E_{NaL}, Na^+ leak current reversal potential; L, length; d, diameter.

Table S7. Passive membrane parameters of cortical cells.

Cell	g_{SD} (nS)	R_{SD} (MΩ)	A_S (μm^2)	A_D (μm^2)	ρ
RS	100.75	9.93	100.07	16011.94	160
EF	100.75	9.93	100.07	16011.94	160
IB	97.69	10.24	100.07	16512.31	165
RIB	94.82	10.55	100.07	17012.68	170
SIB	92.11	10.86	100.07	17513.06	175
ND	100.75	9.93	100.07	16011.94	160
FS	134.33	7.44	100.07	12008.95	120

Abbreviations: R_{SD} is the resistance between the axosomatic and dendritic compartments; ρ is the A_D/A_S ratio.

Table S8. Leak K$^+$ conductance of cortical cells in cortical network model.

Cell	Depolarised	Transitional	Fast slow (delta)	Slow	Hyperpolarised
RS	0.0000043	0.0000143	0.0000173	0.0000173	0.0000243
EF	0.0000043	0.0000128	0.0000128	0.0000128	0.0000243
IB	0.0000093	0.0000193	0.0000223	0.0000223	0.0000243
RIB	0.0000093	0.0000193	0.0000223	0.0000223	0.0000243
ND	0.000002	0.000002	0.000002	0.0000143	0.0000243
FS	0.0000043	0.0000143	0.0000173	0.0000173	0.0000243

Conductances are expressed in mS/cm^2.

Table S9. Leak K$^+$ conductance of all cells in corticothalamic network model.

Cell	Depolarised	Slow (L5/TC init)	Slow (TC init)	Delta	Sleep spindles
RS	0.0000043	0.0000293	0.0000293	0.0000293	0.0000443
EF	0.0000129	0.0000293	0.0000293	0.0000293	0.0000293
IB	0.0000093	0.0000293	0.0000293	0.0000293	0.0000493
RIB	0.0000093	0.0000293	0.0000293	0.0000293	0.0000493
ND	0.00001396	0.0000093	0.0000293	0.0000293	0.0000293
FS	0.0000093	0.0000293	0.0000293	0.0000293	0.0000293
TC_{FO}	0.00133	0.0015	0.0015	0.0019	0.0008
TC_{HO}	0.0044	0.0022	0.0022	0.00305	0.0021
NRT	0.0002	0.0004	0.0004	0.0009	0.0004

Conductances are expressed in mS/cm^2 in cortical cells in μS in thalamic cells.

Table S10. Sets of AMPAR parameters used in the corticothalamic network model.

Synapse	$\bar{g}$ (µS)	E (mV)	α (ms^{-1})	β (ms^{-1})	T_{dur} (ms)	T_{max} (mM)
Cortex	0.001945	0	0.94	0.22	0.55	0.5
NRT	0.04	0	10	3	0.3	0.5
TC	0.034	0	10	3	0.3	0.5

Table S11. Sets of $GABA_AR$ parameters used in the corticothalamic network model.

Synapse	$\bar{g}$ (µS)	E (mV)	α (ms^{-1})	β (ms^{-1})	T_{dur} (ms)	T_{max} (mM)
Cortex	0.068	-80	0.1	0.2	0.8	0.5
NRT	0.8	-70	0.01	0.04	1.5	0.5
TC	1	-70	0.05	2	1.4	0.5

Table S12. Sets of $GABA_BR$ parameters used in the corticothalamic network model.

Synapse	$\bar{g}$ (µS)	k_1 (mM^{-1} ms^{-1})	k_2 (ms^{-1})	k_3 (ms^{-1})	k_4 (ms^{-1})	K_d (mM^4)	T_{dur} (ms)	T_{max} (mM)
Cortex	0.001625	0.18	0.0025	0.19	0.06	17.83	0.8	0.5
TC	0.61	0.2	0.0028	0.28	0.45	100	1.4	0.5

Table S13. Sets of NMDAR parameters used in the corticothalamic network model.

Synapse	$\bar{g}$ (µS)	Mg_o (mM)
Cortex	0.00001275	2
Cx-NRT	0.000003	0.5
TC-NRT	0.000003	0.5
Cx-TC	0.00003	0.5

Supplementary Methods

Model neurons

Thalamic cells were single-compartment Hodgkin-Huxley models described by an equation:

$$C_m \frac{dV_M}{dt} = -G_L(V_M - E_L) - G_{int}(V_M - E_{int}) - G_{syn}(V_M - E_{syn}) - \frac{g_{gap}(V_M - V_N)}{A_M}, \qquad (\boldsymbol{S1})$$

where C_m is the membrane capacitance per unit area in F/cm^2, V_M is the membrane potential in mV, G_L, G_{int}, and G_{syn} are the leak, intrinsic, and synaptic membrane conductances, respectively, in S/cm^2, E_L, E_{int}, and E_{syn} are the reversal potentials for the corresponding conductances in mV, g_{gap} is the gap junction conductance in S, A_M is the membrane area of the cell in cm^2, and V_N is the membrane potential of a neighbouring cell (NRT neurons only) connected by a gap junction (mV).

Cortical cells were Hodgkin-Huxley models with separate axosomatic and dendritic compartments. Equations describing the two corresponding compartments were:

$$C_m \frac{dV_S}{dt} = -G_L(V_S - E_L) - G_{int}(V_S - E_{int}) - G_{syn}(V_S - E_{syn}) - \frac{g_{SD}(V_S - V_D)}{A_S}, \qquad (\boldsymbol{S2})$$

$$C_m \frac{dV_D}{dt} = -G_L(V_D - E_L) - G_{int}(V_D - E_{int}) - G_{syn}(V_D - E_{syn}) - \frac{g_{SD}(V_D - V_S)}{A_D}, \qquad (\boldsymbol{S3})$$

where V_S is the axosomatic membrane potential in mV, V_D is the dendritic membrane potential in mV, g_{SD} is the conductance between the two compartments in S, A_S is the membrane area of the axosomatic compartment in cm^2, and A_D is the membrane area of the dendritic compartment in cm^2.

Intrinsic currents used in TC and NRT neurons and their conductance values are given in the Tables S3 and S4, respectively. Table S5 lists this information for cortical cells. Passive properties common to neuron models are outlined in the Table S6, while those unique to cortical cells are detailed in Table S7.

Membrane currents

Voltage-dependent ion channel currents governing intrinsic membrane potential perturbations were modelled using the Hodgkin-Huxley formalism:

$$I_{int} = \bar{g} m^N h(V_M - E_{int}), \qquad (\boldsymbol{S4})$$

$$\frac{dm}{dt} = \frac{m_\infty - m}{\tau_m}, \qquad (\boldsymbol{S5})$$

$$m_\infty = \frac{\alpha_m}{\alpha_m + \beta_m}, \qquad (\boldsymbol{S6})$$

$$\tau_m = \frac{1}{\alpha_m + \beta_m}, \qquad (\boldsymbol{S7})$$

$$\frac{dh}{dt} = \frac{h_\infty - h}{\tau_h}, \qquad (\boldsymbol{S8})$$

$$h_\infty = \frac{\alpha_h}{\alpha_h + \beta_h}, \qquad (\boldsymbol{S9})$$

$$\tau_h = \frac{1}{\alpha_h + \beta_h}, \qquad (\boldsymbol{S10})$$

where $\bar{g}$ is the maximum conductance in S/cm^2, m and h are state variables describing channel activation and inactivation, respectively, m_∞ and h_∞ are the resting state functions describing activation and inactivation, τ_m and τ_h are state transition time constants for activation and inactivation, α is the forward rate function in ms^{-1}, and β is the backward rate function in ms^{-1}. The Ca^{2+}- and Na^{+}-dependent intrinsic membrane currents followed a similar formalism. These equations apply to intrinsic current descriptions in the Supplementary Appendices A (TC neurons), B (NRT neurons), and C (cortical neurons) unless explicitly stated or replaced by different corresponding equations.

Synaptic currents were described (Appendix D) using a similar formalism with voltage or intracellular ion concentration dependencies replaced by extracellular neurotransmitter concentration dependencies (AMPA, NMDA, $GABA_A$). As for the NMDA channel, the simplification went even further replacing the neurotransmitter concentration by delivering a synaptic event[107]. The NMDA receptor model had neurotransmitter, voltage, and extracellular Mg^{2+} concentration dependencies.

No synaptic plasticity was incorporated into the model. All synapses had the same fixed release probability of 0.8[5,108,109]. Weights associated with each synapse slightly differed among the same type of synapses (standard deviation of 5%) and were pseudo-randomly allocated at the beginning of each simulation. The same applied to synaptic latencies (standard deviation of 20%).

Spontaneous miniature PSP (mPSPs) were generated in all synapses. Their amplitudes were

0.17 mV and 0.083 mV for all cortical AMPA and $GABA_A$ synapses, respectively[110]. mPSPs with 0.5 mV amplitude were used in all thalamic synapses except those of intra-NRT synapses which had an amplitude of 0.25 mV. Both cortical and thalamic mPSPs did not contain NMDA and $GABA_B$ components. mPSPs were generated following an exponential distribution that was dependent on regular synaptic events generated in response to presynaptic action potentials:

$$d(t) = \begin{cases} i_1 n e^{-(t-t_0)}, for\ t - t_0 \leq 1000 \\ i_2 n e^{-(t-t_0)}, for\ t - t_0 > 1000 \end{cases}, \qquad (\boldsymbol{S11})$$

where d is the stimulus delivery delay in ms, t is the time in ms, t_0 is the time of the last presynaptic spike in ms, n is the number of the same type synapses on the cell, i_1 is the average stimulus delivery delay given a single synapse on a neuron immediately following the presynaptic spike, and i_2 is the average stimulus delivery delay given a single synapse on a neuron 1000 ms following the presynaptic spike. For cortical neurons, i_1 and i_2 were equal to 200/3 and 50/3, respectively, whereas for thalamic cells they were 200 and 100, respectively. A mPSP that was still in the queue of delivery at the time when the new presynaptic spike arrived would have its delivery delay reset.

Intracellular concentration dynamics for $[Ca^{2+}]_i$ and $[Na^+]_i$ were modelled by a simple first-order decay (Destexhe et al., 1993a):

$$\frac{d[Ion]_i}{dt} = -\frac{10000 I_{ion}}{ZFd} + \frac{[Ion]_\infty - [Ion]_i}{\tau_D}, \qquad (\boldsymbol{S12})$$

Where $[Ion]_i$ is the intracellular ion concentration in mM, I_{ion} is the sum of all the transmembrane currents carried by the ion in mA/cm^2 (exclude I_{HVA} in TC cells), Z is the valence of the ion, d is the depth of the shell in µm, $[Ion]_\infty$ is the resting intracellular ion concentration in mM, τ_D is the intracellular ion concentration decay time constant in ms. Decay parameters for $[Ca^{2+}]_i$ and $[Na^+]_I$ are in Table S8.

EEG signal

The scalp EEG signal produced by the simulations was estimated based on Bédard et al[111]:

$$V = \frac{1}{4\pi\sigma} \sum_{n=1}^{N} \frac{i_n}{r_n}, \qquad (\boldsymbol{S13})$$

where V is the total sum of N local field potentials (µV) at the surface of the scalp, $\sigma = 0.000355$ mS/µm is the conductivity of the neural tissue[112], i_n is the nth current source in nA, and r_n is the

distance from the electrode to the current source in µm. The excitatory current sources associated with neurons in L2/3 were assumed to have a vertical distance of 351.8 µm, 693.4 µm in L4, 1089.4 µm in L5, and 1597.2 µm in L6[82,113]. The inhibitory current sources were assumed to be 500 µm deeper than the excitatory sources to reflect the somato-dendritic spatial distribution differences of the two synapse types. Neighbouring cells in the same cortical layer were equidistant (20 µm) along the horizontal extent of the layer[114]. The EEG calculations were based only on the synapses located on excitatory neurons[114] since they are the main contributors to cortical local field potentials[115].

Data analyses

Simulated raw EEG traces were filtered using the Butterworth low-pass filter with 40 Hz and 50 Hz passband and stopband frequencies, respectively. Passband ripple and stopband attenuation parameters were set to 0.5 dB and 10 dB, respectively. EEG auto- and cross-correlations were calculated using Matlab's xcorr function. EEG power was calculated using Matlab's fft function.

Membrane potential histograms were produced using 0.2 mV size bins. During cortical slow (<1 Hz) wave simulations Up- and Down-state durations were estimated for each cell individually with the help of membrane potential histograms. The histogram trough between the two bistability peaks was used as a threshold to roughly split the simulated membrane potential into preliminary periods of Up- and Down-states. Preliminary periods of Up-states longer than 100 ms were deemed to be Up-states and the remaining samples were deemed to be Down-states.

APs were assigned to a particular slow (<1 Hz) oscillation Up-state or a delta/sleep spindle cycle by first low-pass filtering the raw EEG trace using the Butterworth filter. Passband ripple and stopband parameters had the same values as those described earlier. Passband and stopband frequencies were 2 Hz and 3 Hz for slow waves, 5 Hz and 7.5 Hz for delta waves, and 20 and 30 Hz for sleep spindles, respectively. Individual EEG oscillation cycle peaks were then identified. Middle points between two neighbouring cycle peaks were deemed to be cycle borders and APs were assigned to their nearest cycles accordingly.

Supplementary Appendices

Appendix A: Intrinsic membrane currents in thalamocortical cell models

This Appendix provides the mathematical descriptions of all intrinsic membrane currents used in TC cell models shown in the equation below:

$$I_{M(TC)} = I_{KL} + I_{NaL} + I_{Na} + I_{K(DR)} + I_T + I_{HVA} + I_h + I_{CAN} + I_{Na(P)} + I_A + I_{K1} + I_{AMPA} + I_{NMDA} + I_{GABAa} + I_{GABAb}. \qquad (\boldsymbol{S14})$$

Their maximum conductances and permeabilities are summarised in Table S3.

The fast transient Na^+ current (I_{Na}) model was adapted from Traub, Wong, Miles and Michelson (1991)[116]:

$$\alpha_m = \frac{0.32(V_M + 28.9)}{1 - e^{-\frac{V_M+28.9}{4}}}, \qquad (\boldsymbol{S15})$$

$$\beta_m = -\frac{0.28(V_M + 1.9)}{1 - e^{\frac{V_M+1.9}{5}}}, \qquad (\boldsymbol{S16})$$

$$\alpha_h = 0.128e^{-\frac{V_M+25}{18}}, \qquad (\boldsymbol{S17})$$

$$\beta_h = \frac{4}{1 + e^{-\frac{V_M+2}{5}}}, \qquad (\boldsymbol{S18})$$

with N = 3 and E_{Na} = 30 mV. The time constants were temperature dependent with the temperature coefficient $q_{10} = 3^{\frac{T-35}{10}}$, where T is the temperature in degrees of Celsius. Only time constants and not amplitudes were temperature-dependent in thalamic cell models.

The persistent delayed rectifier K^+ current ($I_{K(DR)}$) was also adapted from Traub, Wong, Miles and Michelson (1991)[116]:

$$\alpha_m = \frac{0.016(V_M + 2.9)}{1 - e^{-\frac{V_M+2.9}{5}}}, \qquad (\boldsymbol{S19})$$

$$\beta_m = 0.25e^{-\frac{V_M+18}{40}}, \qquad (\boldsymbol{S20})$$

with N = 4 and E_K = -90 mV without the inactivation state h. The temperature coefficient was $q_{10} = 3^{\frac{T-35}{10}}$.

The low voltage activated T-type Ca^{2+} current (I_T) was adapted from Williams, Tóth, Turner, Hughes and Crunelli (1997)[117] and described by these equations:

$$I_T = \bar{g}m^2h(V_M - E_{Ca}), \qquad (\boldsymbol{S21})$$

$$m_\infty = \frac{1}{1 + e^{-\frac{V_M+57}{6.2}}}, \qquad (\boldsymbol{S22})$$

$$\tau_m = \begin{cases} e^{\frac{V_M+220.35}{66.6}}, for\ V_M < -57 \\ 2.44 + 0.02506e^{-0.0984(V_M-3)}, for\ V_M \geq -57 \end{cases}, \qquad (\boldsymbol{S23})$$

$$h_\infty = \frac{1}{1 + e^{\frac{V_M+80.5}{6.3}}}, \qquad (\boldsymbol{S24})$$

$$\tau_h = \begin{cases} e^{\frac{V_M+405.8}{66.6}}, for\ V_M < -77 \\ 7.66 + 0.02868e^{-0.1054(V_M-3)}, for\ V_M \geq -77 \end{cases}, \qquad (\boldsymbol{S25})$$

Where E_{Ca} = 180 mV. Both time constants were temperature dependent with $q_{10} = 3^{\frac{T-35}{10}}$. The model behaviour was matched to the experimental voltage-clamp data in Huguenard and Prince(1992)[118].

The non-inactivating HVA Ca^{2+} channels (I_{HVA}) were modelled as in McCormick and Huguenard (1992)[119] and Kay and Wong (1987)[120] but were adapted so that they did not activate I_{CAN} as observed in Hughes, Cope, Blethyn and Crunelli (2002)[121]. This fact required a separate $[Ca^{2+}]_i$ pool for I_{HVA}. The equations were as follows:

$$I_{HVA} = \bar{P}m^2G(V_M, Ca_o, Ca_i), \qquad (\boldsymbol{S26})$$

$$m_\infty = \frac{1}{1 + e^{-\frac{0.00225F(4.48+V_M)}{R(T-273.15)}}}, \qquad (\boldsymbol{S27})$$

$$\alpha_m = \frac{1.6}{1 + e^{-0.0072(V_M-20)}}, \qquad (\boldsymbol{S28})$$

$$\beta_m = -\frac{0.02(V_M - 7.31)}{1 - e^{\frac{V_M-7.31}{5.36}}}, \qquad (\boldsymbol{S29})$$

$$G(V, Ca_o, Ca_i) = \frac{0.001Z^2F^2V_M\left(Ca_i - Ca_o e^{-\frac{ZFV_M}{R(T+273.15)}}\right)}{1 - e^{-\frac{ZFV_M}{R(T+273.15)}}}, \qquad (\boldsymbol{S30})$$

where Ca_o = 1.5[122-130] and Ca_i are the extracellular and intracellular Ca^{2+} concentrations in mM, respectively, Z = 2 is the valence of calcium ions, F = 96485.309 J is the Faraday constant, R = 8.3144621 J/Kmol is the gas constant, and T is the temperature in degrees of Celsius.. The temperature coefficient was $q_{10} = 3^{\frac{T-21}{10}}$.

I_h was modelled as in Huguenard and McCormick (1992)[131] but was converted into a kinetic

scheme to represent Ca^{2+}-dependence of the channel as in Destexhe, Bal, McCormick and Sejnowski (1996)[132]. The Ca^{2+}-dependence was implemented via Ca^{2+}-binding second messenger protein. The whole model is outlined below:

$$I_h = \bar{g}(o_1 + g_{inc}o_2)(V_M - E_h), \qquad (\boldsymbol{S31})$$

$$c \xleftrightarrow{\alpha_m,\beta_m} o_1, \qquad (\boldsymbol{S32})$$

$$p_0 \xleftrightarrow{k_1,k_2} p_1, \qquad (\boldsymbol{S33})$$

$$o_1 \xleftrightarrow{k_3,k_4} o_2, \qquad (\boldsymbol{S34})$$

$$\alpha_m = \frac{m_\infty}{\tau_m}, \qquad (\boldsymbol{S35})$$

$$\beta_m = \frac{(1 - m_\infty)}{\tau_m}, \qquad (\boldsymbol{S36})$$

$$m_\infty = \frac{1}{1 + e^{-\frac{V_M+75}{5.5}}}, \qquad (\boldsymbol{S37})$$

$$\tau_m = 20 + \frac{1000}{e^{\frac{V_M-89.5}{14.2}} + e^{-\frac{V_M+107}{11.6}}}, \qquad (\boldsymbol{S38})$$

$$k_1 = k_2\left(\frac{Ca_{i(inc)}}{Ca_c}\right)^4, \qquad (\boldsymbol{S39})$$

$$k_3 = \frac{k_4 p_1}{p_c}, \qquad (\boldsymbol{S40})$$

where g_{inc} = 0.5 is the Ca^{2+}-mediated increase in G_h, E_h = -40 mV, c is the proportion of channels in the closed state, o_1 is the proportion of channels in the open protein-unbound state, o_2 is the proportion of channels in the open protein-bound state, p_0 is the proportion of second messenger proteins in the Ca^{2+}-unbound state, p_1 is the proportion of second messenger proteins in the Ca^{2+}-bound state, k_1 and k_2 = 0.00015 are the Ca^{2+}-dependent transition rates between these two protein states in $mM^{-4}ms^{-1}$, k_3 and k_4 = 0.00007 are the Ca^{2+}-bound protein-dependent transition rates between open channel states with different conductances in ms^{-1}, $Ca_{i(inc)}$ is the increase in the $[Ca^{2+}]_i$ relative to the resting value in mM, Ca_c = 0.00085 sets the $Ca_{i(inc)}$ threshold value above which k_1 functions in the superlinear regime (mM), p_c = 0.017 sets the p_1 threshold value above which k_3 exceeds k_4. τ_m depended on temperature with $q_{10} = 3^{\frac{T-36}{10}}$. The model behaviour was tested against the experimental voltage clamp data in McCormick and Pape (1990)[133].

The Ca^{2+}-activated non-specific cation current (I_{CAN}) was implemented using a kinetic scheme

with a Ca^{2+}-binding second messenger molecule[134-136]:

$$I_{CAN} = \bar{g}m^2h(V_M - E_{CAN}), \qquad (\boldsymbol{S41})$$

$$m_\infty = \frac{0.0001(\frac{Ca_i}{0.00045})^2}{0.0001(\frac{Ca_i}{0.00045})^2 + 0.0001}, \qquad (\boldsymbol{S42})$$

$$h_\infty = \frac{1}{(\frac{Ca_i}{0.00036})^{20} + 1}, \qquad (\boldsymbol{S43})$$

$$\tau_h = \max\left(\left\{\frac{1}{0.00019\left(\frac{0.00036}{Ca_i}\right)^{20} + 0.00019}, 0.1\right\}\right), \qquad (\boldsymbol{S44})$$

where E_{CAN} = 0 mV and τ_m = 250 ms. I_{CAN} amplitude and dynamics were constrained by the experimental observations in Hughes, Cope, Blethyn and Crunelli (2002)[121].

$I_{Na(P)}$ was modelled according to Parri and Crunelli (1998)[137] but the activation time constant was adopted from the fast transient Na^+ channels described by Traub, Wong, Miles and Michelson (1991)[116] but hyperpolarised by 37.68 mV:

$$m_\infty = \frac{1}{1 + e^{-\frac{V_M + 53.87)}{8.57}}}, \qquad (\boldsymbol{S45})$$

$$\alpha_m = \frac{0.32(V_M + 66.58)}{1 - e^{-\frac{V_M + 66.58}{4}}}, \qquad (\boldsymbol{S46})$$

$$\beta_m = -\frac{0.28(V_M + 39.58)}{1 - e^{\frac{V_M + 39.58}{5}}}, \qquad (\boldsymbol{S47})$$

with N = 1, $E_{Na(P)}$ = 30 mV, and h being absent. The temperature coefficient was $q_{10} = 3^{\frac{T-35}{10}}$. $I_{Na(P)}$ amplitude was constrained to be within the experimentally observed range reported by Parri and Crunelli (1998)[137].

I_A model was adopted from Huguenard and McCormick (1992)[131] and was constrained to match the voltage clamp data of Huguenard, Coulter and Prince (1991)[138]:

$$I_A = (\bar{g}_1 m_1^4 h_1 + \bar{g}_2 m_2^4 h_2)(V_M - E_A), \qquad (\boldsymbol{S48})$$

$$m_{1\infty} = \frac{1}{1 + e^{-\frac{V_M + 60}{8.5}}}, \qquad (\boldsymbol{S49})$$

$$m_{2\infty} = \frac{1}{1 + e^{-\frac{V_M + 36}{20}}}, \qquad (\boldsymbol{S50})$$

$$\tau_{m1} = \tau_{m2} = \frac{1}{e^{\frac{V_M+35.8}{19.7}} + e^{-\frac{V_M+79.7}{12.7}}}, \qquad (\boldsymbol{S51})$$

$$h_{1\infty} = h_{2\infty} = \frac{1}{1 + e^{\frac{V_M+78}{6}}}, \qquad (\boldsymbol{S52})$$

$$\tau_{h1} = \begin{cases} \frac{1}{e^{\frac{V_M+46}{5}} + e^{-\frac{V_M+238}{37.5}}}, for\ V_M < -63 \\ 19, for\ V_M \geq -63 \end{cases}, \qquad (\boldsymbol{S53})$$

$$\tau_{h2} = \begin{cases} \tau_{h1}, for\ V_M < -73 \\ 60, for\ V_M \geq -73 \end{cases}, \qquad (\boldsymbol{S54})$$

with E_A = -90 mV and $q_{10} = 3^{\frac{T-23}{10}}$.

I_{K1} model was adapted from Huguenard and Prince (1991)[139]:

$$m_{\infty} = \frac{1}{1 + e^{-\frac{V_M+5}{8.6}}}, \qquad (\boldsymbol{S55})$$

with N = 1, E_A = -90 mV, τ_m = 2.5 ms, $q_{10} = 3^{\frac{T-22}{10}}$, and h being absent.

Appendix B: Intrinsic membrane currents in nucleus reticularis thalami cell models

This Appendix provides mathematical descriptions of all intrinsic membrane currents used in NRT cell models shown in the equation below:

$$I_{M(NRT)} = I_{KL} + I_{NaL} + I_{Na} + I_{K(DR)} + I_{Ts} + I_{HVA} + I_h + I_{AHP} + I_{CAN} + I_{Na(P)} + I_{K[Na]} + I_{AMPA} + I_{NMDA} + I_{GABAa} + I_{gap(1,1)} + I_{gap(1,2)} + I_{gap(2,1)} + I_{gap(2,2)}. \qquad (\mathbf{56})$$

Their maximum conductances and permeabilities are summarised in Table S4.

With a few adjustments most of the intrinsic membrane currents in NRT cells were the same as those used in TC cells. They include I_{Na}, $I_{K(DR)}$, I_{HVA}, I_h, I_{CAN}, and $I_{Na(P)}$. I_{Na} voltage dependencies were hyperpolarised relative to TC cells by 8 mV. $I_{K(DR)}$ voltage dependencies were hyperpolarised by 12 mV. With regards to I_h, the following parameters were changed: g_{inc} = 2, k_4 = 0.00007 ms^{-1}, Ca_c = 0.00175 mM, and p_c = 0.017.

The model for the slow T-type Ca^{2+} current (I_{Ts}) was described in Huguenard and Prince (1992)[118] with time constants adopted from Destexhe, Contreras, Steriade, Sejnowski and Huguenard (1996)[140]:

$$E_{Ca} = \frac{1000R(T + 273.15)}{2F} log_{10}\left(\frac{Ca_o}{Ca_i}\right), \qquad (\boldsymbol{S57})$$

$$m_\infty = \frac{1}{1 + e^{-\frac{V_M+50}{7.4}}}, \qquad (\boldsymbol{S58})$$

$$\tau_m = 3 + \frac{1}{e^{\frac{V_M+25}{10}} + e^{-\frac{V_M+100}{15}}}, \qquad (\boldsymbol{S59})$$

$$h_\infty = \frac{1}{1 + e^{\frac{V_M+78}{5}}}, \qquad (\boldsymbol{S60})$$

$$\tau_h = 85 + \frac{1}{e^{\frac{V_M+46}{4}} + e^{-\frac{V_M+405}{50}}}, \qquad (\boldsymbol{S61})$$

with N = 2, Ca_o = 1.5 mM, and $q_{10} = 3^{\frac{T-24}{10}}$.

The I_{AHP} model was outlined in Xia, Fakler, Rivard, Wayman, Johnson-Pais, Keen, Ishii, Hirschberg, Bond, Lutsenko, Maylie and Adelman (1998)[141] and calibrated by data in Cueni, Canepari, Lujan, Emmenegger, Watanabe, Bond, Franken, Adelman and Luthi (2008)[142]:

$$I_{AHP} = (\bar{g}_1 m_{1\infty} + \bar{g}_2 m_{2\infty})(V_M - E_{CAN}), \qquad (\boldsymbol{S62})$$

$$m_{1\infty} = \frac{1}{(\frac{Ca_{EC50,1}}{Ca_{i(inc)}})^{5.3} + 1}, \qquad (\boldsymbol{S63})$$

$$m_{2\infty} = \frac{1}{(\frac{Ca_{EC50,2}}{Ca_{i(inc)}})^{5.3} + 1}, \qquad (\boldsymbol{S64})$$

with E_A = -90 mV, $Ca_{EC50,\,1}$ = 0.001, and $Ca_{EC50,\,2}$ = 0.00032 are $[Ca^{2+}]_i$ of the half-maximal response (mM) for the two components, τ_{m1} = 15 ms, τ_{m2} = 830 ms, $q_{10} = 3^{\frac{T-34.25}{10}}$, and h being absent.

The Ca^{2+}-activated non-specific cation current (I_{CAN}) was implemented following the scheme outlined for the TC cells but with a few differences. Modified equations are shown below:

$$I_{CAN} = \bar{g}m^2(V_M - E_{CAN}), \qquad (\boldsymbol{S65})$$

$$m_{\infty} = \frac{0.000004(\frac{Ca_i}{0.00045})^2}{0.000004(\frac{Ca_i}{0.00045})^2 + 0.000004}, \qquad (\boldsymbol{S66})$$

$$\tau_m = \frac{1}{0.000004(\frac{Ca_i}{0.00045})^2 + 0.000004}, \qquad (\boldsymbol{S67})$$

where E_{CAN} = 0 mV.

$I_{K[Na]}$ was taken from Bischoff, Vogel and Safronov (1998)[143]:

$$m = \frac{1}{1 + (\frac{38.7}{Na_i})^{3.5}}, \qquad (\boldsymbol{S68})$$

With N = 1, $E_{K[Na]}$ = -90 mV, $q_{10} = 2.3^{\frac{T-37}{10}}$ for the amplitude, and h being absent.

Appendix C: Intrinsic membrane currents in neocortical cell models

This Appendix provides mathematical descriptions of all intrinsic membrane currents used in the cortical cell models shown in the equations below:

$$I_S = I_{KL} + I_{NaL} + I_{Na} + I_{K(DR)} + I_{Na(P)} + I_{K[Na]} + I_{GABAa} + I_{GABAb} + I_{DS}, \qquad (\boldsymbol{S69})$$

$$I_D = I_{KL} + I_{NaL} + I_{Na} + I_A + I_M + I_{fAHP} + I_{sAHP} + I_h + I_{Na(P)} + I_{K[Na]} + I_T + I_{HVA} + I_{AMPA} + I_{NMDA} + I_{SD}, \qquad (\boldsymbol{S70})$$

Most of the models of cortical currents were used previously in Mainen and Sejnowski (1996)[144]. Their maximum conductances and permeabilities are summarised in Table S5.

I_{Na} is included in both axosomatic and dendritic compartments and was originally taken from Mainen and Sejnowski (1996)[144]:

$$\alpha_m = \frac{0.182(V_M + 25)}{1 - e^{-\frac{V_M+25}{9}}}, \qquad (\boldsymbol{S71})$$

$$\beta_m = -\frac{0.124(V_M + 25)}{1 - e^{\frac{V_M+25}{9}}}, \qquad (\boldsymbol{S72})$$

$$\alpha_h = \frac{0.024(V_M + 40)}{1 - e^{-\frac{V_M+40}{5}}}, \qquad (\boldsymbol{S73})$$

$$\beta_h = -\frac{0.0091(V_M + 65)}{1 - e^{-\frac{V_M+65}{5}}}, \qquad (\boldsymbol{S74})$$

$$\tau_h = \frac{1}{1 + e^{\frac{V_M+55}{6.2}}}, \qquad (\boldsymbol{S75})$$

with N = 3 and E_{Na} = 60 mV.

$I_{K(DR)}$ model was used only in the axosomatic compartment and was adopted from the same source:

$$\alpha_m = \frac{0.02(V_M - 25)}{1 - e^{-\frac{V_M-25}{9}}}, \qquad (\boldsymbol{S76})$$

$$\beta_m = -\frac{0.002(V_M - 25)}{1 - e^{\frac{V_M-25}{9}}}, \qquad (\boldsymbol{S77})$$

with N = 1 and h being absent. Amplitudes and time constants of both I_{Na} and $I_{K(DR)}$ increased and decreased with temperature, respectively. The temperature factor was $q_{10} = 2.3^{\frac{T-23}{10}}$.

$I_{Na(P)}$ was expressed in both compartments and adopted from Mainen and Sejnowski (19960[144] with the time constant taken from Timofeev, Grenier, Bazhenov, Sejnowski and Steriade (2000)[145]:

$$m_{\infty} = \frac{1}{1 + e^{-\frac{V_M + 42}{5}}}, \qquad (\boldsymbol{S78})$$

with N = 1, $E_{Na(P)}$ = 60 mV, τ_m = 0.05 ms, $q_{10} = 2.3^{\frac{T-36}{10}}$ for the amplitude and the time constant, and h being absent.

$I_{K[Na]}$ was also expressed in both compartments and was taken from Bischoff, Vogel and Safronov (1998)[143]:

$$m = \frac{1}{1 + (\frac{38.7}{Na_i})^{3.5}}, \qquad (\boldsymbol{S79})$$

with N = 1, $E_{K[Na]}$ = -90 mV $q_{10} = 2.3^{\frac{T-37}{10}}$ for the amplitude, and h being absent.

I_A was expressed in the dendritic compartment, modelled according to Keren, Peled and Korngreen (2005)[146] and constrained against the experimental data of Korngreen and Sakmann (2000)[147]:

$$m_{\infty} = \frac{1}{1 + e^{-\frac{V_M + 47}{29}}}, \qquad (\boldsymbol{S80})$$

$$\tau_m = 0.34 + 0.92e^{-(\frac{V_M + 71}{59})^2}, \qquad (\boldsymbol{S81})$$

$$h_{\infty} = \frac{1}{1 + e^{\frac{V_M + 66}{10}}}, \qquad (\boldsymbol{S82})$$

$$\tau_h = 8 + 49e^{-(\frac{V_M + 73}{23})^2}, \qquad (\boldsymbol{S83})$$

with N = 4, E_A = -90 mV, and $q_{10} = 2.3^{\frac{T-21}{10}}$ for the amplitude and time constants.

Similarly, I_M was localised within the dendritic compartment and modelled according to Mainen and Sejnowski (1996)[144] and Yamada, Koch and Adams (1989)[148]:

$$\alpha_m = \frac{0.0001(V_M + 30)}{1 - e^{-\frac{V_M + 30}{9}}}, \qquad (\boldsymbol{S84})$$

$$\beta_m = -\frac{0.0001(V_M + 30)}{1 - e^{\frac{V_M + 30}{9}}}, \qquad (\boldsymbol{S85})$$

with N = 1, E_A = -90 mV, $q_{10} = 2.3^{\frac{T-23}{10}}$ for the amplitude and the time constant, and h being absent.

I_{fAHP} was expressed in the dendritic compartment only and adopted from Mainen and Sejnowski (1996)[144] with α = 10Ca_i, β = 0.02, with N = 1, E_A = -90 mV, $q_{10} = 2.3^{\frac{T-23}{10}}$ for the amplitude and the time constant, and h being absent. Meanwhile I_{sAHP} was based on a model derived in the context of non-cortical cells[141,142] with equations being the same as in NRT cells (see Equations S62-64). Changes were: $\bar{g}_2$ = 0.000001-0.00145 S/cm^2. I_{sAHP} was present only in dendritic compartments of ND cells.

I_h was taken from Keren, Peled and Korngreen (2005)[146] with the Ca^{2+}-dependence modelled similarly to thalamic cells[132]. The equations were also the same except for m_∞, τ_m, and k_1 which were:

$$m_\infty = \frac{1}{1 + e^{-\frac{V_M+91}{6}}}, \qquad (\boldsymbol{S86})$$

$$\tau_m = \frac{1}{0.0004e^{-0.025V_M}+0.088e^{0.062V_M}}, \qquad (\boldsymbol{S87})$$

$$k_1 = k_2(\frac{Ca_i}{Ca_c})^4. \qquad (\boldsymbol{S88})$$

Other parameters were $E_{K[Na]}$ = -30 mV, k_2 = 0.00015 mM^{-4}ms^{-1}, k_4 = 0.00007 ms^{-1}, Ca_c = 0.0015 mM, p_c = 0.017, $q_{10} = 3.5^{\frac{T-36}{10}}$ for the time constant only, and h being absent.

I_T was based on Destexhe, Neubig, Ulrich and Huguenardv (1998)[149] and described by Goldman-Hodgkin-Katz equations:

$$I_T = \bar{P}m^2hG(V_M, Ca_o, Ca_i), \qquad (\boldsymbol{S89})$$

$$m_\infty = \frac{1}{1 + e^{-\frac{V_M+57}{6.2}}}, \qquad (\boldsymbol{S90})$$

$$\tau_m = 0.612 + \frac{1}{e^{-\frac{V_M+132}{16.7}}+e^{\frac{V_M+16.8}{18.2}}}, \qquad (\boldsymbol{S91})$$

$$h_\infty = \frac{1}{1 + e^{\frac{V_M+81}{4}}}, \qquad (\boldsymbol{S92})$$

$$\tau_h = \begin{cases} e^{\frac{V_M+467}{66.6}}, for\ V_M \le -80 \\ 28 + e^{-\frac{V_M+22}{10.5}}, for\ V_M > -80 \end{cases}, \qquad (\boldsymbol{S93})$$

where $\overline{P}$ is the maximum membrane permeability to Ca^{2+} in cm/s ($\overline{P}_{RS,EF}$ = 0.000001 and $\overline{P}_{IB,RIB,ND}$ = 0.00001). Both time constants were temperature dependent with $q_{10} = 3^{\frac{T-24}{10}}$. I_T was absent in FS cells.

I_{HVA} was modelled according to Mainen and Sejnowski (1996)[144]:

$$\alpha_m = \frac{0.055(V_M + 27)}{1 - e^{-\frac{V_M+27}{3.8}}}, \qquad (\boldsymbol{S94})$$

$$\beta_m = 0.94e^{-\frac{V_M+75}{17}}, \qquad (\boldsymbol{S95})$$

$$\alpha_h = 0.000457e^{-\frac{V_M+13}{50}}, \qquad (\boldsymbol{S96})$$

$$\beta_h = \frac{0.0065}{1 + e^{-\frac{V_M+15}{28}}}, \qquad (\boldsymbol{S97})$$

with N = 2, E_{HVA} = 140 mV, Ca_o = 1.5 mM, and $q_{10} = 2.3^{\frac{T-23}{10}}$ for the amplitude and time.

Appendix D: Synaptic membrane current models

This appendix provides the mathematical descriptions of synaptic current models and their parameters used in the corticothalamic network model.

Except the NMDA component, AMPA, $GABA_A$, and $GABA_B$ postsynaptic currents were modelled based on a simplifying assumption of the neurotransmitter concentration dynamics in the synaptic cleft as a unitary amplitude pulse as described in Destexhe, Mainen and Sejnowski (1994)[150]:

$$I_{AMPA/GABA_A} = \bar{g}m(V_M - E_{AMPA/GABA_A}), \qquad (\boldsymbol{S98})$$

$$m = \begin{cases} m_\infty + (m(t_0) - m_\infty)e^{-\frac{t-t_0}{\tau_m}}, for\ t - t_0 \le T_{dur} \\ m(t_0 + T_{dur})e^{-\beta(t-t_0-T_{dur})}, for\ t - t_0 > T_{dur} \end{cases}, \qquad (\boldsymbol{S98})$$

$$m_\infty = \frac{\alpha T_{max}}{\alpha T_{max} + \beta}, \qquad (\boldsymbol{S100})$$

$$\tau_m = \frac{1}{\alpha T_{max} + \beta}, \qquad (\boldsymbol{S101})$$

where $I_{AMPA/GABAa}$ is the postsynaptic current in nA, $\bar{g}$ is the maximal conductance in µS, t_0 is the onset time of the neurotransmitter pulse (ms), T_{dur} is the duration of the neurotransmitter pulse (ms), T_{max} is the amplitude of the pulse in mM. Tables S10 and S11 summarise AMPAR and $GABA_A$R parameter sets used in this model.

Thalamic and cortical postsynaptic $GABA_B$ currents were based on the same simplifying solution but involving a second messenger protein as outlined in Destexhe, Bal, McCormick and Sejnowski (1996)[132] and Thomson and Destexhe (1990)[40]:

$$\frac{dR}{dt} = k_1 T_{max}(1 - R) - k_2 R, \qquad (\boldsymbol{S102})$$

$$\frac{dP}{dt} = k_3 R - k_4 P, \qquad (\boldsymbol{S103})$$

$$I_{GABA_B} = \bar{g}m\frac{P^4}{P^4 + K_d}(V_M - E_{GABA_B}), \qquad (\boldsymbol{S104})$$

where E_{GABAb} = -90 mV, R is the fraction of activated receptor, P is the concentration of activated second messenger protein in mM, K_d is the dissociation constant of the binding of the activated protein on the K^+ channels mediating the $GABA_B$ postsynaptic current in mM^4, k_1 ($mM^{-1}ms^{-1}$) and k_2 (ms^{-1}) are the forward and backward receptor state transition rates,

respectively, whereas k_3 (ms^{-1}) and k_4 (ms^{-1}) are second messenger protein activation and inactivation rates, respectively. The transmitter T_{max} is only present for a limited period T_{dur}. The sets of $GABA_BR$ parameters are outlined in Table S12.

The NMDA postsynaptic current model in the thalamus and the cortex was the most complex of all other synaptic channels used here and was based on the work presented in Moradi, Moradi, Ganjkhani, Hajihasani, Gharibzadeh and Kaka (2013)[107] but excluding short-term depression. The following is the outline:

$$I_{NMDA} = \bar{g}(f_{VI} + f_{VD})(w_C C + w_B B - A)Mg(V_M - E_{NMDA}), \qquad (\boldsymbol{S105})$$

$$\frac{\partial f_{VD}}{\partial t} = -\frac{f_{VI}(w_C C + w_B B)(f_{VD,\infty} - f_{VD})}{\tau_g}, \qquad (\boldsymbol{S106})$$

$$\tau_g = \frac{7}{q_{10,g}}, \qquad (\boldsymbol{S107})$$

$$q_{10,g} = 1.52^{\frac{(T-26)}{10}}, \qquad (\boldsymbol{S108})$$

$$g_{VD,\infty} = k(V_M - V_0), \qquad (\boldsymbol{S109})$$

$$\frac{dA}{dt} = -\frac{A}{\tau_A}, \qquad (\boldsymbol{S110})$$

$$\tau_A = \frac{\tau_{A,0} + a_A e^{-\lambda_A V_M}}{q_{10,A}}, \qquad (\boldsymbol{S111})$$

$$q_{10,A} = 2.2^{\frac{(T-35)}{10}}, \qquad (\boldsymbol{S112})$$

$$\frac{dB}{dt} = -\frac{B}{\tau_B}, \qquad (\boldsymbol{S113})$$

$$\tau_B = \frac{\tau_{B,0} + a_B(1 - e^{-\lambda_B V_M})}{q_{10,B}}, \qquad (\boldsymbol{S114})$$

$$q_{10,B} = 3.68^{\frac{(T-35)}{10}}, \qquad (\boldsymbol{S115})$$

$$\frac{dC}{dt} = -\frac{C}{\tau_C}, \qquad (\boldsymbol{S116})$$

$$\tau_C = \frac{\tau_{C,0} + a_C(1 - e^{-\lambda_C V_M})}{q_{10,C}}, \qquad (\boldsymbol{S117})$$

$$q_{10,C} = 2.65^{\frac{(T-35)}{10}}, \qquad (\boldsymbol{S118})$$

$$Mg = \frac{1}{1 + \frac{Mg_o}{Mg_{IC50}} e^{-\frac{0.001Z\delta F V_M}{R(T+273.15)}}}, \qquad (\boldsymbol{S119})$$

where E_{NMDA} = -0.7 mV, f_{VI} = 0.5 and f_{VD} are the voltage-independent and voltage-dependent channel conductance components in fractions, respectively, $f_{VD,\infty}$ is the resting voltage-dependent conductance function (μS), V_0 = -100 mV is the baseline V_M at which the f_{VD} = 0, k =0.007 mV^{-1} is the factor relating V_M change to the f_{VD}, τ_g is the voltage-dependent conductance transition time constant (ms), A is the channel activation state dependent on the neurotransmitter, B and C are the deactivation states, w_B = 0.65 and w_C = 0.35 set the proportions of the two inactivation terms ($w_B + w_C = 1$), Mg determines the Mg^{2+} block, τ_A, τ_B, and τ_C are the activation and the two deactivation time constants (ms), $\tau_{A,0}$ = 3 ms, $\tau_{B,0}$ = 25.057 ms, and $\tau_{C,0}$ = 232.27 ms are initial time constants (ms) at V_m = 0 mV, a_A = 1, a_B = 2.2364, and a_C = 43.495 are tuning factors, λ_A = 1, λ_B = 0.0243, and λ_C = 0.01 are decay constants, Mg_o is the extracellular Mg^{2+} concentration in mM, Mg_{IC50} = 4.1 mM is the 50% Mg^{2+} inhibition concentration in mM at V_m = 0 mV, Z = 2 is the valence of Mg^{2+}, and δ = 0.8 is the relative electrical distance of the binding site of Mg^{2+} from the outside of the membrane. The sets of NMDAR parameters are outlined in Table S13.

Supplementary References

1. Chen W, Zhang JJ, Hu GY, Wu CP. Electrophysiological and morphological properties of pyramidal and nonpyramidal neurons in the cat motor cortex in vitro. *Neuroscience*. Jul 1996;73(1):39-55.
2. Lorincz ML, Gunner D, Bao Y, et al. A distinct class of slow (~0.2-2 Hz) intrinsically bursting layer 5 pyramidal neurons determines UP/DOWN state dynamics in the neocortex. *The Journal of neuroscience : the official journal of the Society for Neuroscience*. Apr 8 2015;35(14):5442-58. doi:10.1523/JNEUROSCI.3603-14.2015
3. Blethyn KL, Hughes SW, Tóth TI, Cope DW, Crunelli V. Neuronal basis of the slow (<1 Hz) oscillation in neurons of the nucleus reticularis thalami in vitro. *The Journal of neuroscience : the official journal of the Society for Neuroscience*. Mar 1 2006;26(9):2474-86. doi:10.1523/jneurosci.3607-05.2006
4. Beierlein M, Gibson JR, Connors BW. Two dynamically distinct inhibitory networks in layer 4 of the neocortex. *Journal of neurophysiology*. 2003;90(5):2987-3000.
5. Feldmeyer D. Excitatory neuronal connectivity in the barrel cortex. *Frontiers in neuroanatomy*. 2012;6:24. doi:10.3389/fnana.2012.00024
6. Hooks BM, Hires SA, Zhang YX, et al. Laminar analysis of excitatory local circuits in vibrissal motor and sensory cortical areas. *PLoS biology*. 2011;9(1):e1000572. doi:10.1371/journal.pbio.1000572
7. Kumar P, Ohana O. Inter- and intralaminar subcircuits of excitatory and inhibitory neurons in layer 6a of the rat barrel cortex. *Journal of neurophysiology*. Oct 2008;100(4):1909-22. doi:10.1152/jn.90684.2008
8. Lee CC, Sherman SM. Modulator property of the intrinsic cortical projection from layer 6 to layer 4. *Frontiers in systems neuroscience*. 2009;3:3.
9. Lee CC, Lam Y-W, Sherman SM. Intracortical convergence of layer 6 neurons. *Neuroreport*. 2012;23(12):736.
10. Lefort S, Tomm C, Sarria J-CF, Petersen CC. The excitatory neuronal network of the C2 barrel column in mouse primary somatosensory cortex. *Neuron*. 2009;61(2):301-316.
11. Markram H, Muller E, Ramaswamy S, et al. Reconstruction and Simulation of Neocortical Microcircuitry. *Cell*. Oct 8 2015;163(2):456-92. doi:10.1016/j.cell.2015.09.029
12. Oberlaender M, Boudewijns ZS, Kleele T, Mansvelder HD, Sakmann B, de Kock CP. Three-dimensional axon morphologies of individual layer 5 neurons indicate cell type-specific intracortical pathways for whisker motion and touch. *Proceedings of the National Academy of Sciences*. 2011;108(10):4188-4193.
13. Crochet S, Petersen CC. Correlating whisker behavior with membrane potential in barrel cortex of awake mice. *Nat Neurosci*. May 2006;9(5):608-10. doi:10.1038/nn1690
14. Pichon F, Nikonenko I, Kraftsik R, Welker E. Intracortical connectivity of layer VI pyramidal neurons in the somatosensory cortex of normal and barrelless mice. *The European journal of neuroscience*. Mar 2012;35(6):855-69. doi:10.1111/j.1460-9568.2012.08011.x
15. Thomson AM. Neocortical layer 6, a review. *Frontiers in neuroanatomy*. 2010;4:13. doi:10.3389/fnana.2010.00013
16. Binzegger T, Douglas RJ, Martin KA. A quantitative map of the circuit of cat primary visual cortex. *The Journal of neuroscience : the official journal of the Society for Neuroscience*. Sep 29 2004;24(39):8441-53. doi:10.1523/JNEUROSCI.1400-04.2004
17. George D, Hawkins J. Towards a Mathematical Theory of Cortical Micro-circuits. *PLoS Comput Biol*. 2009;5(10):e1000532. doi:10.1371/journal.pcbi.1000532
18. Fino E, Yuste R. Dense inhibitory connectivity in neocortex. *Neuron*. Mar 24 2011;69(6):1188-203. doi:10.1016/j.neuron.2011.02.025
19. Levy RB, Reyes AD. Spatial profile of excitatory and inhibitory synaptic connectivity in mouse primary auditory cortex. *The Journal of neuroscience : the official journal of the Society for Neuroscience*. Apr 18 2012;32(16):5609-19. doi:10.1523/JNEUROSCI.5158-11.2012

20. Fino E, Packer AM, Yuste R. The logic of inhibitory connectivity in the neocortex. *The Neuroscientist : a review journal bringing neurobiology, neurology and psychiatry*. Jun 2013;19(3):228-37. doi:10.1177/1073858412456743
21. Feldmeyer D, Roth A, Sakmann B. Monosynaptic connections between pairs of spiny stellate cells in layer 4 and pyramidal cells in layer 5A indicate that lemniscal and paralemniscal afferent pathways converge in the infragranular somatosensory cortex. *The Journal of neuroscience*. 2005;25(13):3423-3431.
22. Schubert D, Kötter R, Luhmann H, Staiger J. Morphology, electrophysiology and functional input connectivity of pyramidal neurons characterizes a genuine layer Va in the primary somatosensory cortex. *Cerebral cortex*. 2006;16(2):223-236.
23. Tanaka YR, Tanaka YH, Konno M, et al. Local connections of excitatory neurons to corticothalamic neurons in the rat barrel cortex. *The Journal of neuroscience : the official journal of the Society for Neuroscience*. Dec 14 2011;31(50):18223-36. doi:10.1523/JNEUROSCI.3139-11.2011
24. Brill J, Huguenard JR. Robust short-latency perisomatic inhibition onto neocortical pyramidal cells detected by laser-scanning photostimulation. *The Journal of neuroscience : the official journal of the Society for Neuroscience*. Jun 10 2009;29(23):7413-23. doi:10.1523/JNEUROSCI.6098-08.2009
25. Markram H. A network of tufted layer 5 pyramidal neurons. *Cerebral cortex*. 1997;7(6):523-533.
26. Markram H, Lübke J, Frotscher M, Roth A, Sakmann B. Physiology and anatomy of synaptic connections between thick tufted pyramidal neurones in the developing rat neocortex. *The Journal of physiology*. 1997;500(Pt 2):409.
27. West DC, Mercer A, Kirchhecker S, Morris OT, Thomson AM. Layer 6 cortico-thalamic pyramidal cells preferentially innervate interneurons and generate facilitating EPSPs. *Cerebral cortex*. Feb 2006;16(2):200-11. doi:10.1093/cercor/bhi098
28. Maffei A, Nelson SB, Turrigiano GG. Selective reconfiguration of layer 4 visual cortical circuitry by visual deprivation. *Nat Neurosci*. Dec 2004;7(12):1353-9. doi:10.1038/nn1351
29. Myme CI, Sugino K, Turrigiano GG, Nelson SB. The NMDA-to-AMPA ratio at synapses onto layer 2/3 pyramidal neurons is conserved across prefrontal and visual cortices. *Journal of neurophysiology*. Aug 2003;90(2):771-9. doi:10.1152/jn.00070.2003
30. Umemiya M, Senda M, Murphy TH. Behaviour of NMDA and AMPA receptor-mediated miniature EPSCs at rat cortical neuron synapses identified by calcium imaging. *The Journal of physiology*. 1999;521(1):113-122.
31. Watt AJ, van Rossum MC, MacLeod KM, Nelson SB, Turrigiano GG. Activity coregulates quantal AMPA and NMDA currents at neocortical synapses. *Neuron*. 2000;26(3):659-670.
32. Watt AJ, Sjöström PJ, Häusser M, Nelson SB, Turrigiano GG. A proportional but slower NMDA potentiation follows AMPA potentiation in LTP. *Nature neuroscience*. 2004;7(5):518-524.
33. Gentet LJ, Avermann M, Matyas F, Staiger JF, Petersen CC. Membrane potential dynamics of GABAergic neurons in the barrel cortex of behaving mice. *Neuron*. Feb 11 2010;65(3):422-35. doi:10.1016/j.neuron.2010.01.006
34. Galarreta M, Hestrin S. Properties of GABAA receptors underlying inhibitory synaptic currents in neocortical pyramidal neurons. *The Journal of neuroscience*. 1997;17(19):7220-7227.
35. Hájos N, Nusser Z, Rancz EA, Freund TF, Mody I. Cell type-and synapse-specific variability in synaptic GABAA receptor occupancy. *European Journal of Neuroscience*. 2000;12(3):810-818.
36. Hutcheon B, Morley P, Poulter MO. Developmental change in GABAA receptor desensitization kinetics and its role in synapse function in rat cortical neurons. *The Journal of physiology*. 2000;522(1):3-17.
37. Perrais D, Ropert N. Effect of zolpidem on miniature IPSCs and occupancy of postsynaptic GABAA receptors in central synapses. *The Journal of neuroscience*. 1999;19(2):578-588.
38. Salin PA, Prince DA. Spontaneous GABAA receptor-mediated inhibitory currents in adult rat somatosensory cortex. *Journal of neurophysiology*. 1996;75(4):1573-1588.

39. Thomson AM, West DC, Hahn J, Deuchars J. Single axon IPSPs elicited in pyramidal cells by three classes of interneurones in slices of rat neocortex. *The Journal of physiology*. 1996;496(Pt 1):81.
40. Thomson AM, Destexhe A. Dual intracellular recordings and computational models of slow inhibitory postsynaptic potentials in rat neocortical and hippocampal slices. *Neuroscience*. 1999;92(4):1193-1215.
41. Chattopadhyaya B, Di Cristo G, Higashiyama H, et al. Experience and activity-dependent maturation of perisomatic GABAergic innervation in primary visual cortex during a postnatal critical period. *The Journal of neuroscience*. 2004;24(43):9598-9611.
42. Petreanu L, Mao T, Sternson SM, Svoboda K. The subcellular organization of neocortical excitatory connections. *Nature*. Feb 26 2009;457(7233):1142-5. doi:10.1038/nature07709
43. Lam YW, Sherman SM. Mapping by laser photostimulation of connections between the thalamic reticular and ventral posterior lateral nuclei in the rat. *Journal of neurophysiology*. Oct 2005;94(4):2472-83. doi:10.1152/jn.00206.2005
44. Lam YW, Sherman SM. Different topography of the reticulothalmic inputs to first- and higher-order somatosensory thalamic relays revealed using photostimulation. *Journal of neurophysiology*. Nov 2007;98(5):2903-9. doi:10.1152/jn.00782.2007
45. Lam YW, Nelson CS, Sherman SM. Mapping of the functional interconnections between thalamic reticular neurons using photostimulation. *Journal of neurophysiology*. Nov 2006;96(5):2593-600. doi:10.1152/jn.00555.2006
46. Sanchez-Vives MV, Bal T, McCormick DA. Inhibitory Interactions between Perigeniculate GABAergic Neurons. *The Journal of Neuroscience*. November 15, 1997 1997;17(22):8894-8908.
47. Zhang SJ, Huguenard JR, Prince DA. GABAA Receptor-Mediated Cl− Currents in Rat Thalamic Reticular and Relay Neurons. *Journal of neurophysiology*. November 1, 1997 1997;78(5):2280-2286.
48. Huntsman MM, Porcello DM, Homanics GE, DeLorey TM, Huguenard JR. Reciprocal Inhibitory Connections and Network Synchrony in the Mammalian Thalamus. *Science (New York, NY)*. January 22, 1999 1999;283(5401):541-543. doi:10.1126/science.283.5401.541
49. Huntsman MM, Huguenard JR. Nucleus-Specific Differences in GABAA-Receptor-Mediated Inhibition Are Enhanced During Thalamic Development. *Journal of neurophysiology*. January 1, 2000 2000;83(1):350-358.
50. Ulrich D, Huguenard JR. Purinergic inhibition of GABA and glutamate release in the thalamus: Implications for thalamic network activity. *Neuron*. 10// 1995;15(4):909-918. doi:http://dx.doi.org/10.1016/0896-6273(95)90181-7
51. Ulrich D, Huguenard JR. GABAB receptor-mediated responses in GABAergic projection neurones of rat nucleus reticularis thalami in vitro. *The Journal of physiology*. June 15, 1996 1996;493(Pt 3):845-854.
52. Huntsman MM, Huguenard JR. Fast IPSCs in rat thalamic reticular nucleus require the GABAA receptor beta1 subunit. *The Journal of physiology*. Apr 15 2006;572(Pt 2):459-75. doi:10.1113/jphysiol.2006.106617
53. Mozrzymas JW, Barberis A, Vicini S. GABAergic currents in RT and VB thalamic nuclei follow kinetic pattern of alpha3- and alpha1-subunit-containing GABAA receptors. *The European journal of neuroscience*. Aug 2007;26(3):657-65. doi:10.1111/j.1460-9568.2007.05693.x
54. Ulrich D, Huguenard JR. Nucleus-Specific Chloride Homeostasis in Rat Thalamus. *The Journal of Neuroscience*. April 1, 1997 1997;17(7):2348-2354.
55. Bazhenov M, Timofeev I, Steriade M, Sejnowski TJ. Self-sustained rhythmic activity in the thalamic reticular nucleus mediated by depolarizing GABAA receptor potentials. 10.1038/5729. *Nat Neurosci*. 02//print 1999;2(2):168-174.
56. Shu Y, McCormick DA. Inhibitory Interactions Between Ferret Thalamic Reticular Neurons. *Journal of neurophysiology*. May 1, 2002 2002;87(5):2571-2576. doi:10.1152/jn.00850.2001
57. Zhang L, Jones EG. Corticothalamic Inhibition in the Thalamic Reticular Nucleus. *Journal of neurophysiology*. February 1, 2004 2004;91(2):759-766. doi:10.1152/jn.00624.2003

58. Sun YG, Wu CS, Renger JJ, Uebele VN, Lu HC, Beierlein M. GABAergic synaptic transmission triggers action potentials in thalamic reticular nucleus neurons. *The Journal of neuroscience : the official journal of the Society for Neuroscience*. Jun 6 2012;32(23):7782-90. doi:10.1523/JNEUROSCI.0839-12.2012
59. Jones EG. *The thalamus*. 2 ed. Cambridge University Press; 2007:1708.
60. Warren RA, Agmon A, Jones EG. Oscillatory synaptic interactions between ventroposterior and reticular neurons in mouse thalamus in vitro. *Journal of neurophysiology*. October 1, 1994 1994;72(4):1993-2003.
61. Kim U, McCormick DA. The Functional Influence of Burst and Tonic Firing Mode on Synaptic Interactions in the Thalamus. *The Journal of Neuroscience*. November 15, 1998 1998;18(22):9500-9516.
62. Gentet LJ, Ulrich D. Strong, reliable and precise synaptic connections between thalamic relay cells and neurones of the nucleus reticularis in juvenile rats. *The Journal of physiology*. February 1, 2003 2003;546(3):801-811. doi:10.1113/jphysiol.2002.032730
63. Zhang Z, Liu CH, Yu YQ, Fujimoto K, Chan YS, He J. Corticofugal projection inhibits the auditory thalamus through the thalamic reticular nucleus. *Journal of neurophysiology*. Jun 2008;99(6):2938-45. doi:10.1152/jn.00002.2008
64. Bal T, von Krosigk M, McCormick DA. Synaptic and membrane mechanisms underlying synchronized oscillations in the ferret lateral geniculate nucleus in vitro. *The Journal of physiology*. Mar 15 1995;483 (Pt 3):641-63.
65. Evrard A, Ropert N. Early Development of the Thalamic Inhibitory Feedback Loop in the Primary Somatosensory System of the Newborn Mice. *The Journal of Neuroscience*. August 5, 2009 2009;29(31):9930-9940. doi:10.1523/jneurosci.1671-09.2009
66. Lam YW, Sherman SM. Functional organization of the thalamic input to the thalamic reticular nucleus. *The Journal of neuroscience : the official journal of the Society for Neuroscience*. May 4 2011;31(18):6791-9. doi:10.1523/JNEUROSCI.3073-10.2011
67. Herkenham M. Laminar organization of thalamic projections to the rat neocortex. *Science (New York, NY)*. Feb 1 1980;207(4430):532-5.
68. Jones EG. The thalamic matrix and thalamocortical synchrony. *Trends in neurosciences*. 2001;24(10):595-601.
69. Lee CC, Sherman SM. Synaptic properties of thalamic and intracortical inputs to layer 4 of the first- and higher-order cortical areas in the auditory and somatosensory systems. *Journal of neurophysiology*. Jul 2008;100(1):317-26. doi:10.1152/jn.90391.2008
70. Beierlein M, Fall CP, Rinzel J, Yuste R. Thalamocortical bursts trigger recurrent activity in neocortical networks: layer 4 as a frequency-dependent gate. *J Neurosci*. // 2002;22:9885-9894.
71. Gabernet L, Jadhav SP, Feldman DE, Carandini M, Scanziani M. Somatosensory integration controlled by dynamic thalamocortical feed-forward inhibition. *Neuron*. Oct 20 2005;48(2):315-27. doi:10.1016/j.neuron.2005.09.022
72. Cruikshank SJ, Lewis TJ, Connors BW. Synaptic basis for intense thalamocortical activation of feedforward inhibitory cells in neocortex. *Nat Neurosci*. Apr 2007;10(4):462-8. doi:10.1038/nn1861
73. Tan Z, Hu H, Huang ZJ, Agmon A. Robust but delayed thalamocortical activation of dendritic-targeting inhibitory interneurons. *Proceedings of the National Academy of Sciences of the United States of America*. Feb 12 2008;105(6):2187-92. doi:10.1073/pnas.0710628105
74. Hull C, Isaacson JS, Scanziani M. Postsynaptic mechanisms govern the differential excitation of cortical neurons by thalamic inputs. *The Journal of neuroscience : the official journal of the Society for Neuroscience*. Jul 15 2009;29(28):9127-36. doi:10.1523/JNEUROSCI.5971-08.2009
75. Cruikshank SJ, Urabe H, Nurmikko AV, Connors BW. Pathway-specific feedforward circuits between thalamus and neocortex revealed by selective optical stimulation of axons. *Neuron*. Jan 28 2010;65(2):230-45. doi:10.1016/j.neuron.2009.12.025

76. Kimura F, Itami C, Ikezoe K, et al. Fast activation of feedforward inhibitory neurons from thalamic input and its relevance to the regulation of spike sequences in the barrel cortex. *The Journal of physiology*. Aug 1 2010;588(Pt 15):2769-87. doi:10.1113/jphysiol.2010.188177
77. Meyer HS, Wimmer VC, Hemberger M, et al. Cell type-specific thalamic innervation in a column of rat vibrissal cortex. *Cerebral cortex*. Oct 2010;20(10):2287-303. doi:10.1093/cercor/bhq069
78. Wimmer VC, Bruno RM, de Kock CP, Kuner T, Sakmann B. Dimensions of a projection column and architecture of VPM and POm axons in rat vibrissal cortex. *Cerebral cortex*. Oct 2010;20(10):2265-76. doi:10.1093/cercor/bhq068
79. Bagnall MW, Hull C, Bushong EA, Ellisman MH, Scanziani M. Multiple clusters of release sites formed by individual thalamic afferents onto cortical interneurons ensure reliable transmission. *Neuron*. Jul 14 2011;71(1):180-94. doi:10.1016/j.neuron.2011.05.032
80. Viaene AN, Petrof I, Sherman M. Properties of the thalamic projection from the posterior medial nucleus to primary and secondary somatosensory cortices in the mouse. *Proceedings of the National Academy of Sciences of the United States of America*. Nov 2011;108(44):18156-18161. doi:10.1073/pnas.1114828108
81. Viaene AN, Petrof I, Sherman SM. Synaptic properties of thalamic input to layers 2/3 and 4 of primary somatosensory and auditory cortices. *Journal of neurophysiology*. Jan 2011;105(1):279-92. doi:10.1152/jn.00747.2010
82. Oberlaender M, de Kock CP, Bruno RM, et al. Cell type-specific three-dimensional structure of thalamocortical circuits in a column of rat vibrissal cortex. *Cerebral cortex*. Oct 2012;22(10):2375-91. doi:10.1093/cercor/bhr317
83. Ohno S, Kuramoto E, Furuta T, et al. A morphological analysis of thalamocortical axon fibers of rat posterior thalamic nuclei: a single neuron tracing study with viral vectors. *Cerebral cortex*. Dec 2012;22(12):2840-57. doi:10.1093/cercor/bhr356
84. Constantinople CM, Bruno RM. Deep Cortical Layers Are Activated Directly by Thalamus. *Science (New York, NY)*. Jun 2013;340(6140):1591-1594. doi:10.1126/science.1236425
85. Herkenham M. The afferent and efferent connections of the ventromedial thalamic nucleus in the rat. *The Journal of Comparative Neurology*. 1979;183(3):487-517. doi:10.1002/cne.901830304
86. Arbuthnott GW, MacLeod NK, Maxwell DJ, Wright AK. Distribution and synaptic contacts of the cortical terminals arising from neurons in the rat ventromedial thalamic nucleus. *Neuroscience*. 1990;38(1):47-60.
87. Rubio-Garrido P, Perez-de-Manzo F, Porrero C, Galazo MJ, Clasca F. Thalamic Input to Distal Apical Dendrites in Neocortical Layer 1 Is Massive and Highly Convergent. *Cerebral cortex*. Oct 2009;19(10):2380-2395. doi:10.1093/cercor/bhn259
88. Cruikshank SJ, Ahmed OJ, Stevens TR, et al. Thalamic control of layer 1 circuits in prefrontal cortex. *The Journal of neuroscience : the official journal of the Society for Neuroscience*. Dec 5 2012;32(49):17813-23. doi:10.1523/JNEUROSCI.3231-12.2012
89. Berendse HW, Groenewegen HJ. Restricted cortical termination fields of the midline and intralaminar thalamic nuclei in the rat. *Neuroscience*. 1991;42(1):73-102.
90. Deschênes M, Veinante P, Zhang Z-W. The organization of corticothalamic projections: reciprocity versus parity. *Brain research reviews*. 1998;28(3):286-308.
91. Golshani P, Liu X-B, Jones EG. Differences in quantal amplitude reflect GluR4-subunit number at corticothalamic synapses on two populations of thalamic neurons. *Proceedings of the National Academy of Sciences*. 2001;98(7):4172-4177.
92. Gentet LJ, Ulrich D. Electrophysiological characterization of synaptic connections between layer VI cortical cells and neurons of the nucleus reticularis thalami in juvenile rats. *European Journal of Neuroscience*. 2004;19(3):625-633.
93. Alexander G, Fisher T, Godwin D. Differential response dynamics of corticothalamic glutamatergic synapses in the lateral geniculate nucleus and thalamic reticular nucleus. *Neuroscience*. 2006;137(2):367-372.

94. Landisman CE, Connors BW. VPM and PoM nuclei of the rat somatosensory thalamus: intrinsic neuronal properties and corticothalamic feedback. *Cerebral cortex*. Dec 2007;17(12):2853-65. doi:10.1093/cercor/bhm025
95. Miyata M, Imoto K. Contrary roles of kainate receptors in transmitter release at corticothalamic synapses onto thalamic relay and reticular neurons. *The Journal of physiology*. 2009;587(5):999-1012.
96. Lam YW, Sherman SM. Functional organization of the somatosensory cortical layer 6 feedback to the thalamus. *Cerebral cortex*. Jan 2010;20(1):13-24. doi:10.1093/cercor/bhp077
97. Lacey CJ, Bryant A, Brill J, Huguenard JR. Enhanced NMDA receptor-dependent thalamic excitation and network oscillations in stargazer mice. *The Journal of neuroscience : the official journal of the Society for Neuroscience*. Aug 8 2012;32(32):11067-81. doi:10.1523/JNEUROSCI.5604-11.2012
98. Reichova I, Sherman SM. Somatosensory corticothalamic projections: distinguishing drivers from modulators. *Journal of neurophysiology*. 2004;92(4):2185-2197.
99. Hsu CL, Yang HW, Yen CT, Min MY. Comparison of synaptic transmission and plasticity between sensory and cortical synapses on relay neurons in the ventrobasal nucleus of the rat thalamus. *The Journal of physiology*. 2010;588(22):4347-4363.
100. Paz JT, Bryant AS, Peng K, et al. A new mode of corticothalamic transmission revealed in the Gria4−/− model of absence epilepsy. *Nature Neuroscience*. 2011;14(9):1167-1173. doi:10.1038/nn.2896
101. Jones EG, Powell TP. An electron microscopic study of the mode of termination of cortico-thalamic fibres within the sensory relay nuclei of the thalamus. *Proceedings of the Royal Society of London Series B, Biological sciences*. Mar 11 1969;172(1027):173-85.
102. Majorossy K, Kiss A. Specific patterns of neuron arrangement and of synaptic articulation in the medial geniculate body. *Exp Brain Res*. Aug 27 1976;26(1):1-17.
103. Somogyi G, Hajdu F, Tombol T. Ultrastructure of the anterior ventral and anterior medial nuclei of the cat thalamus. *Exp Brain Res*. Mar 15 1978;31(3):417-31.
104. Liu XB, Honda CN, Jones EG. Distribution of four types of synapse on physiologically identified relay neurons in the ventral posterior thalamic nucleus of the cat. *J Comp Neurol*. Jan 30 1995;352(1):69-91. doi:10.1002/cne.903520106
105. Granseth B, Lindström S. Unitary EPSCs of corticogeniculate fibers in the rat dorsal lateral geniculate nucleus in vitro. *Journal of neurophysiology*. 2003;89(6):2952-2960.
106. Mease RA, Krieger P, Groh A. Cortical control of adaptation and sensory relay mode in the thalamus. *Proceedings of the National Academy of Sciences*. 2014;111(18):6798-6803.
107. Moradi K, Moradi K, Ganjkhani M, Hajihasani M, Gharibzadeh S, Kaka G. A fast model of voltage-dependent NMDA receptors. *Journal of computational neuroscience*. Jun 2013;34(3):521-31. doi:10.1007/s10827-012-0434-4
108. Castro-Alamancos MA, Connors BW. Distinct forms of short-term plasticity at excitatory synapses of hippocampus and neocortex. *Proceedings of the National Academy of Sciences*. April 15, 1997 1997;94(8):4161-4166.
109. Silver RA, Lübke J, Sakmann B, Feldmeyer D. High-Probability Uniquantal Transmission at Excitatory Synapses in Barrel Cortex. *Science (New York, NY)*. 2003;302(5652):1981-1984. doi:10.1126/science.1087160
110. Dervinis M, Major G. Novel method for reliably measuring spontaneous postsynaptic potentials/currents in whole- cell patch clamp recordings in the central nervous system. *bioRxiv*. 2022:2022.03.20.485046. doi:10.1101/2022.03.20.485046
111. Bédard C, Kröger H, Destexhe A. Modeling extracellular field potentials and the frequency-filtering properties of extracellular space. *Biophysical journal*. 2004;86(3):1829-1842.
112. Ranck Jr JB. Specific impedance of rabbit cerebral cortex. *Experimental Neurology*. 2// 1963;7(2):144-152. doi:http://dx.doi.org/10.1016/S0014-4886(63)80005-9

113. Egger R, Narayanan RT, Helmstaedter M, de Kock CP, Oberlaender M. 3D reconstruction and standardization of the rat vibrissal cortex for precise registration of single neuron morphology. *PLoS Comput Biol*. 2012;8(12):e1002837. doi:10.1371/journal.pcbi.1002837
114. Destexhe A, Contreras D, Steriade M. Mechanisms Underlying the Synchronizing Action of Corticothalamic Feedback Through Inhibition of Thalamic Relay Cells. *Journal of neurophysiology*. February 1, 1998 1998;79(2):999-1016.
115. Bazhenov M, Lonjers P, Skorheim S, Bedard C, Dstexhe A. Non-homogeneous extracellular resistivity affects the current-source density profiles of up-down state oscillations. *Philosophical transactions Series A, Mathematical, physical, and engineering sciences*. Oct 13 2011;369(1952):3802-19. doi:10.1098/rsta.2011.0119
116. Traub RD, Wong R, Miles R, Michelson H. A model of a CA3 hippocampal pyramidal neuron incorporating voltage-clamp data on intrinsic conductances. *Journal of neurophysiology*. 1991;66(2):635-650.
117. Williams SR, Tóth TI, Turner JP, Hughes SW, Crunelli V. The 'window' component of the low threshold Ca2+ current produces input signal amplification and bistability in cat and rat thalamocortical neurones. *The Journal of physiology*. December 15, 1997 1997;505(Pt 3):689-705.
118. Huguenard J, Prince D. A novel T-type current underlies prolonged Ca(2+)-dependent burst firing in GABAergic neurons of rat thalamic reticular nucleus. *The Journal of Neuroscience*. October 1, 1992 1992;12(10):3804-3817.
119. McCormick DA, Huguenard JR. A model of the electrophysiological properties of thalamocortical relay neurons. *Journal of neurophysiology*. October 1, 1992 1992;68(4):1384-1400.
120. Kay A, Wong R. Calcium current activation kinetics in isolated pyramidal neurones of the Ca1 region of the mature guinea-pig hippocampus. *The Journal of physiology*. 1987;392:603.
121. Hughes SW, Cope DW, Blethyn KL, Crunelli V. Cellular Mechanisms of the Slow (<1 Hz) Oscillation in Thalamocortical Neurons In Vitro. *Neuron*. 2002;33(6):947-958.
122. Nicholson C, Bruggencate Gt, Steinberg R, Stöckle H. Calcium modulation in brain extracellular microenvironment demonstrated with ion-selective micropipette. *Proceedings of the National Academy of Sciences*. 1977;74(3):1287-1290.
123. Nicholson C, Ten Bruggencate G, Stockle H, Steinberg R. Calcium and potassium changes in extracellular microenvironment of cat cerebellar cortex. *Journal of neurophysiology*. 1978;41(4):1026-1039.
124. Somjen GG. Stimulus-evoked and seizure-related responses of extracellular calcium activity in spinal cord compared to those in cerebral cortex. *Journal of neurophysiology*. 1980;44(4):617-632.
125. Benninger C, Kadis J, Prince D. Extracellular calcium and potassium changes in hippocampal slices. *Brain research*. 1980;187(1):165-182.
126. Pumain R, Kurcewicz I, Louvel J. Fast extracellular calcium transients: involvement in epileptic processes. *Science (New York, NY)*. 1983;222(4620):177-179.
127. Pumain R, Heinemann U. Stimulus-and amino acid-induced calcium and potassium changes in rat neocortex. *Journal of neurophysiology*. 1985;53(1):1-16.
128. Heinemann U, Stabel J, Rausche G. Activity-dependent ionic changes and neuronal plasticity in rat hippocampus. *Progress in brain research*. 1990;83:197-214.
129. Lücke A, Köhling R, Straub H, Moskopp D, Wassmann H, Speckmann E-J. Changes of extracellular calcium concentration induced by application of excitatory amino acids in the human neocortex in vitro. *Brain research*. 1995;671(2):222-226.
130. Massimini M, Amzica F. Extracellular calcium fluctuations and intracellular potentials in the cortex during the slow sleep oscillation. *Journal of neurophysiology*. 2001;85(3):1346-1350.
131. Huguenard JR, McCormick DA. Simulation of the currents involved in rhythmic oscillations in thalamic relay neurons. *Journal of neurophysiology*. October 1, 1992 1992;68(4):1373-1383.
132. Destexhe A, Bal T, McCormick DA, Sejnowski TJ. Ionic mechanisms underlying synchronized oscillations and propagating waves in a model of ferret thalamic slices. *Journal of neurophysiology*. September 1, 1996 1996;76(3):2049-2070.

133. McCormick DA, Pape HC. Properties of a hyperpolarization-activated cation current and its role in rhythmic oscillation in thalamic relay neurones. *The Journal of physiology*. December 1, 1990 1990;431(1):291-318.
134. Destexhe A, Contreras D, Sejnowski TJ, Steriade M. A model of spindle rhythmicity in the isolated thalamic reticular nucleus. *Journal of neurophysiology*. August 1, 1994 1994;72(2):803-818.
135. Kolaj M, Zhang L, Renaud LP. Novel coupling between TRPC-like and KNa channels modulates low threshold spike-induced afterpotentials in rat thalamic midline neurons. *Neuropharmacology*. 2014/11/01/ 2014;86:88-96. doi:https://doi.org/10.1016/j.neuropharm.2014.06.023
136. Zhang L, Kolaj M, Renaud LP. Endocannabinoid 2-AG and intracellular cannabinoid receptors modulate a low-threshold calcium spike-induced slow depolarizing afterpotential in rat thalamic paraventricular nucleus neurons. *Neuroscience*. 2016/05/13/ 2016;322:308-319. doi:https://doi.org/10.1016/j.neuroscience.2016.02.047
137. Parri HR, Crunelli V. Sodium Current in Rat and Cat Thalamocortical Neurons: Role of a Non-Inactivating Component in Tonic and Burst Firing. *The Journal of Neuroscience*. February 1, 1998 1998;18(3):854-867.
138. Huguenard JR, Coulter DA, Prince DA. A fast transient potassium current in thalamic relay neurons: kinetics of activation and inactivation. *Journal of neurophysiology*. October 1, 1991 1991;66(4):1304-1315.
139. Huguenard JR, Prince DA. Slow inactivation of a TEA-sensitive K current in acutely isolated rat thalamic relay neurons. *Journal of neurophysiology*. October 1, 1991 1991;66(4):1316-1328.
140. Destexhe A, Contreras D, Steriade M, Sejnowski TJ, Huguenard JR. In vivo, in vitro, and computational analysis of dendritic calcium currents in thalamic reticular neurons. *The Journal of neuroscience*. 1996;16(1):169-185.
141. Xia XM, Fakler B, Rivard A, et al. Mechanism of calcium gating in small-conductance calcium-activated potassium channels. 10.1038/26758. *Nature*. 10/01/print 1998;395(6701):503-507.
142. Cueni L, Canepari M, Lujan R, et al. T-type Ca2+ channels, SK2 channels and SERCAs gate sleep-related oscillations in thalamic dendrites. *Nat Neurosci*. Jun 2008;11(6):683-92. doi:10.1038/nn.2124
143. Bischoff U, Vogel W, Safronov BV. Na+-activated K+ channels in small dorsal root ganglion neurones of rat. *The Journal of physiology*. 1998;510(3):743-754.
144. Mainen ZF, Sejnowski TJ. Influence of dendritic structure on firing pattern in model neocortical neurons. 10.1038/382363a0. *Nature*. 07/25/print 1996;382(6589):363-366.
145. Timofeev I, Grenier F, Bazhenov M, Sejnowski TJ, Steriade M. Origin of slow cortical oscillations in deafferented cortical slabs. 10.1093/cercor/10.12.1185. *Cereb Cortex*. // 2000;10:1185-1199.
146. Keren N, Peled N, Korngreen A. Constraining compartmental models using multiple voltage recordings and genetic algorithms. *Journal of neurophysiology*. 2005;94(6):3730-3742.
147. Korngreen A, Sakmann B. Voltage-gated K+ channels in layer 5 neocortical pyramidal neurones from young rats: subtypes and gradients. *The Journal of physiology*. June 15, 2000 2000;525(3):621-639. doi:10.1111/j.1469-7793.2000.00621.x
148. Yamada WM, Koch C, Adams PR. Multiple channels and calcium dynamics. MIT press; 1989:97-133.
149. Destexhe A, Neubig M, Ulrich D, Huguenard J. Dendritic Low-Threshold Calcium Currents in Thalamic Relay Cells. *The Journal of Neuroscience*. May 15, 1998 1998;18(10):3574-3588.
150. Destexhe A, Mainen ZF, Sejnowski TJ. An efficient method for computing synaptic conductances based on a kinetic model of receptor binding. *Neural Computation*. 1994;6:14-18.
